# Distinct transcriptional responses to fludioxonil in *Aspergillus fumigatus* and its Δ*tcs*C and Δ*skn*7 mutants reveal a crucial role for Skn7 in the cell wall reorganizations triggered by this antifungal

**DOI:** 10.1186/s12864-023-09777-5

**Published:** 2023-11-14

**Authors:** Sebastian Schruefer, Annica Pschibul, Sarah Sze Wah Wong, Tongta Sae-Ong, Thomas Wolf, Sascha Schäuble, Gianni Panagiotou, Axel A. Brakhage, Vishukumar Aimanianda, Olaf Kniemeyer, Frank Ebel

**Affiliations:** 1 Institute for Infectious Diseases and Zoonoses, Ludwig-Maximilians-University, Munich, Germany; 2 Molecular and Applied Microbiology, Leibniz Institute for Natural Product Research and Infection Biology - Hans Knöll Institute, Jena, Germany; 3 UMR2000, Molecular Mycology Unit, Mycology Department, Institut Pasteur, Université Paris Cité, CNRS, Paris, France; 4 Microbiome Dynamics, Leibniz Institute for Natural Product Research and Infection Biology - Hans Knöll Institute, Jena, Germany; 5 Institute of Microbiology, Friedrich Schiller University, Jena, Germany; 6 Institut Pasteur, Université Paris Cité, Immunobiology of Aspergillus, Mycology Department, Paris, France

**Keywords:** *Aspergillus fumigatus*, Fludioxonil, HOG pathway, Skn7, TcsC, Multistep phosphorelay

## Abstract

**Background:**

*Aspergillus fumigatus* is a major fungal pathogen that causes severe problems due to its increasing resistance to many therapeutic agents. Fludioxonil is a compound that triggers a lethal activation of the fungal-specific High Osmolarity Glycerol pathway. Its pronounced antifungal activity against *A. fumigatus* and other pathogenic molds renders this agent an attractive lead substance for the development of new therapeutics. The group III hydride histidine kinase TcsC and its downstream target Skn7 are key elements of the multistep phosphorelay that represents the initial section of the High Osmolarity Glycerol pathway. Loss of *tcs*C results in resistance to fludioxonil, whereas a Δ*skn*7 mutant is partially, but not completely resistant.

**Results:**

In this study, we compared the fludioxonil-induced transcriptional responses in the Δ*tcs*C and Δ*skn*7 mutant and their parental *A. fumigatus* strain. The number of differentially expressed genes correlates well with the susceptibility level of the individual strains. The wild type and, to a lesser extend also the Δ*skn*7 mutant, showed a multi-faceted stress response involving genes linked to ribosomal and peroxisomal function, iron homeostasis and oxidative stress. A marked difference between the sensitive wild type and the largely resistant Δ*skn*7 mutant was evident for many cell wall-related genes and in particular those involved in the biosynthesis of chitin. Biochemical data corroborate this differential gene expression that does not occur in response to hyperosmotic stress.

**Conclusions:**

Our data reveal that fludioxonil induces a strong and TcsC-dependent stress that affects many aspects of the cellular machinery. The data also demonstrate a link between Skn7 and the cell wall reorganizations that foster the characteristic ballooning and the subsequent lysis of fludioxonil-treated cells.

**Supplementary Information:**

The online version contains supplementary material available at 10.1186/s12864-023-09777-5.

## Background

*Aspergillus fumigatus* is an opportunistic pathogen that can cause life-threatening infections in severely immunocompromised patients, e.g., after bone marrow or organ transplantation. More recently, invasive aspergillosis was also identified as a severe complication of certain viral infections, e.g., COVID-19 and influenza [1]. The severity of the *A. fumigatus*-induced diseases contrasts with the limited therapeutic options, and this is compounded by an increasing resistance of *A. fumigatus* isolates to azoles, which represent the most potent class of antifungals. New therapeutic strategies and agents are therefore urgently needed to improve this alarming situation.

Pyrrolnitrin is a natural antifungal that was first isolated from *Pseudomonas pyrrocinia* [2] and has a strong antifungal activity against *A. fumigatus* [3]. Pyrrolnitrin targets fungal-specific group III hybrid histidine kinases (HHKs) that in turn activate the HOG pathway, a central stress pathway of fungal cells. Fludioxonil is a chemical derivative of pyrrolnitrin that is utilized in agriculture to combat plant fungal diseases and although in use for more than 30-years now, this agent has not been associated with large-scale resistance problems yet [4]. Hence, it appears as if resistance to fludioxonil comes with a severe disadvantage, at least under environmental conditions. Both, fludioxonil and pyrrolnitrin trigger a dramatic swelling of the target cells, a process that finally culminates in lysis [5]. The characteristic ballooning of the affected fungal elements is a hallmark of the antifungal activity of fludioxonil, but other features are also evident and include reorganizations of the cell wall, a disturbed mitotic process characterized by the disappearance of the NimA kinase from the septal pores, the subsequent closure of these passages and an increased number of nuclei per cell [6]. Fludioxonil employs a similar deleterious activity on *A. nidulans*, but the cellular consequences of this process are currently less well defined than for *A. fumigatus*. The homologous group III HHKs, TcsC in *A. fumigatus* and NikA in *A. nidulans*, play a crucial and non-redundant role in the antifungal processes triggered by fludioxonil and pyrrolnitrin [3, 5, 7]. If activated, both HHKs interact with their downstream target, the histidine-containing phosphotransfer proteins Ypd1 in *A. fumigatus* and YpdA in *A. nidulans*. This, in turn, modulates the activities of the two response regulators SskA / Skn7 in *A. fumigatus* and SskA / SrrA in *A. nidulans*. Both proteins are involved in the antifungal effect of fludioxonil, but in *A. fumigatus* Skn7 is more important in this context [8]. Phosphorylation of SskA initiates the MAP kinase cascade of the HOG pathway that terminates in SakA, which upon activation translocates to the nucleus. Skn7 is per se a nuclear protein in *A. fumigatus* [9]. In *S. cerevisiae,* Skn7p was shown to act as a transcription factor and had been implicated in processes such as cell wall organization and oxidative stress response [10, 11]. In *A. fumigatus,* loss of *skn*7 renders the fungus more sensitive to oxidative stress, but concurrently causes a pronounced resistance to fludioxonil that is accompanied by distinct cell wall rearrangements in this mutant [8, 9].

The distinct levels of sensitivity to fludioxonil found in the Δ*tcs*C and the Δ*skn*7 mutant as well as in their parental *A. fumigatus* strain AfS35 prompted us to compare the transcriptome of these three strains in the presence and absence of fludioxonil. Our aim was to correlate data on differential gene expression with the individual levels of resistance, but also to identify regulatory patterns that provide new insights into the complex mode of action employed by fludioxonil.

## Results

**Fig. 1:**
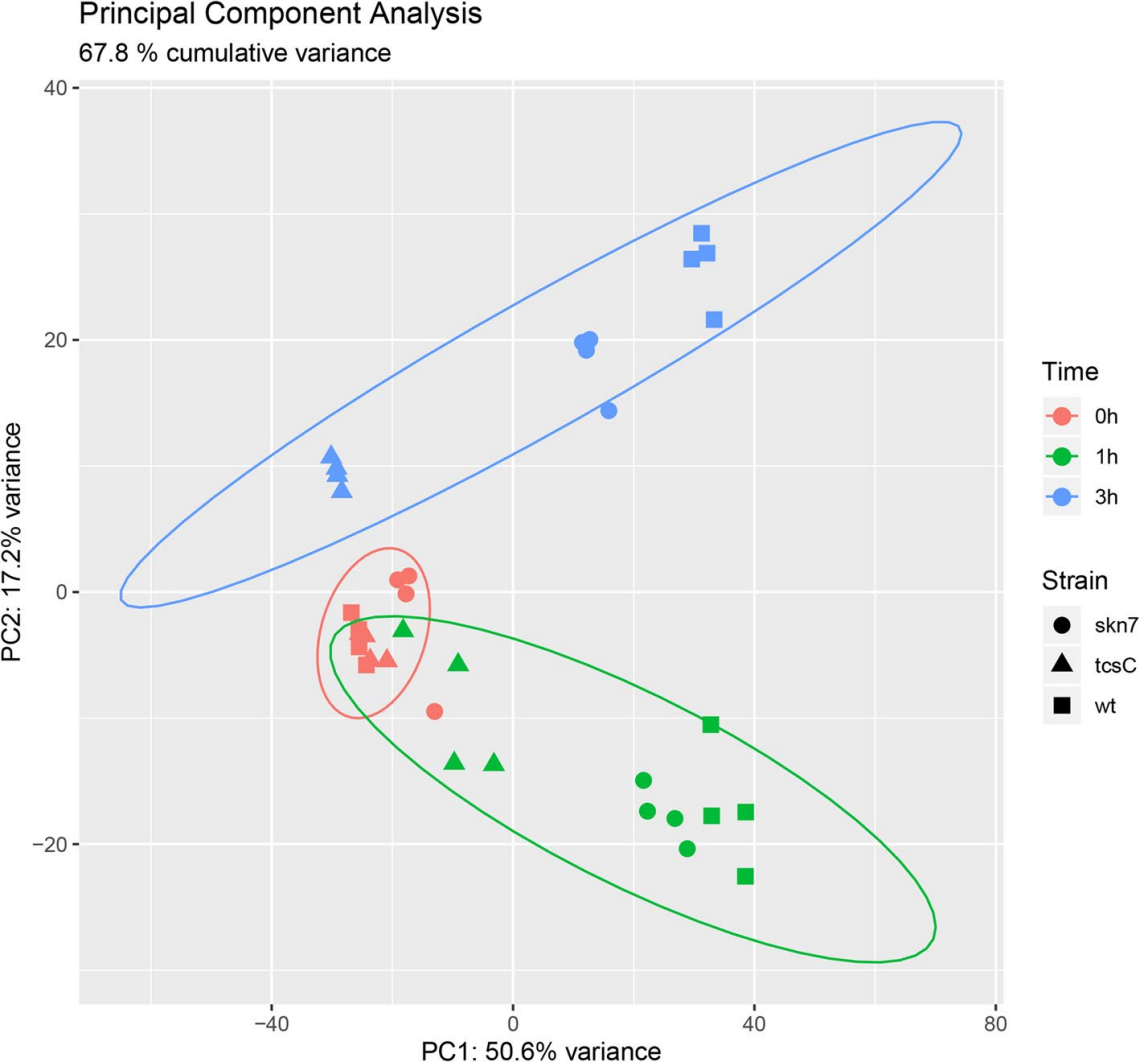
Principle component analysis (PCA) plot of all samples at the three timepoints. *wt* Wild type strain AfS35, *skn7* ∆*skn*7 mutant, *tcsC* ∆*tcs*C mutant. The controls (t = 0 h; in red) are close to each other, whereas the fludioxonil-treated samples at the time point 1 h (green) and especially at the time point 3 h (blue) show an increasing separation by strain

To investigate the role of Skn7 and TcsC in the transcriptional response of *A. fumigatus* to fludioxonil, we performed an RNA-seq analysis of the Δ*tcs*C mutant, the Δ*skn*7 mutant and their parental strain AfS35 before as well as 1 and 3 h after addition of fludioxonil (final concentration 2 µg/ml). These time points were chosen, since the impact of fludioxonil on *A. fumigatus* develops slowly and takes several hours before phenotypic changes are evident [5]. This resulted in a total of 36 samples and FASTQ files, respectively. We acquired 4.8 M to 22.4 M high-quality 75 bp single-end reads per FASTQ file (average about 11 M per sample, about 400 M in total), to reach 10.9-fold to 51.4-fold genome and 16.2-fold to 75.6-fold transcriptome (exome) coverage per sample. The sample replicates were very similar with a correlation coefficient of R > 0.982 for all pairwise correlations. In the principal component analysis (PCA) plot, all untreated samples (t = 0 h) are tightly grouped, whereas the fludioxonil-treated samples of the three strains were increasingly separated at time point 1 h (green) and especially after 3 h (blue) (Fig. 1). At these time points, the Δ*tcs*C mutant differed clearly from the other two strains.

**Fig. 2:**
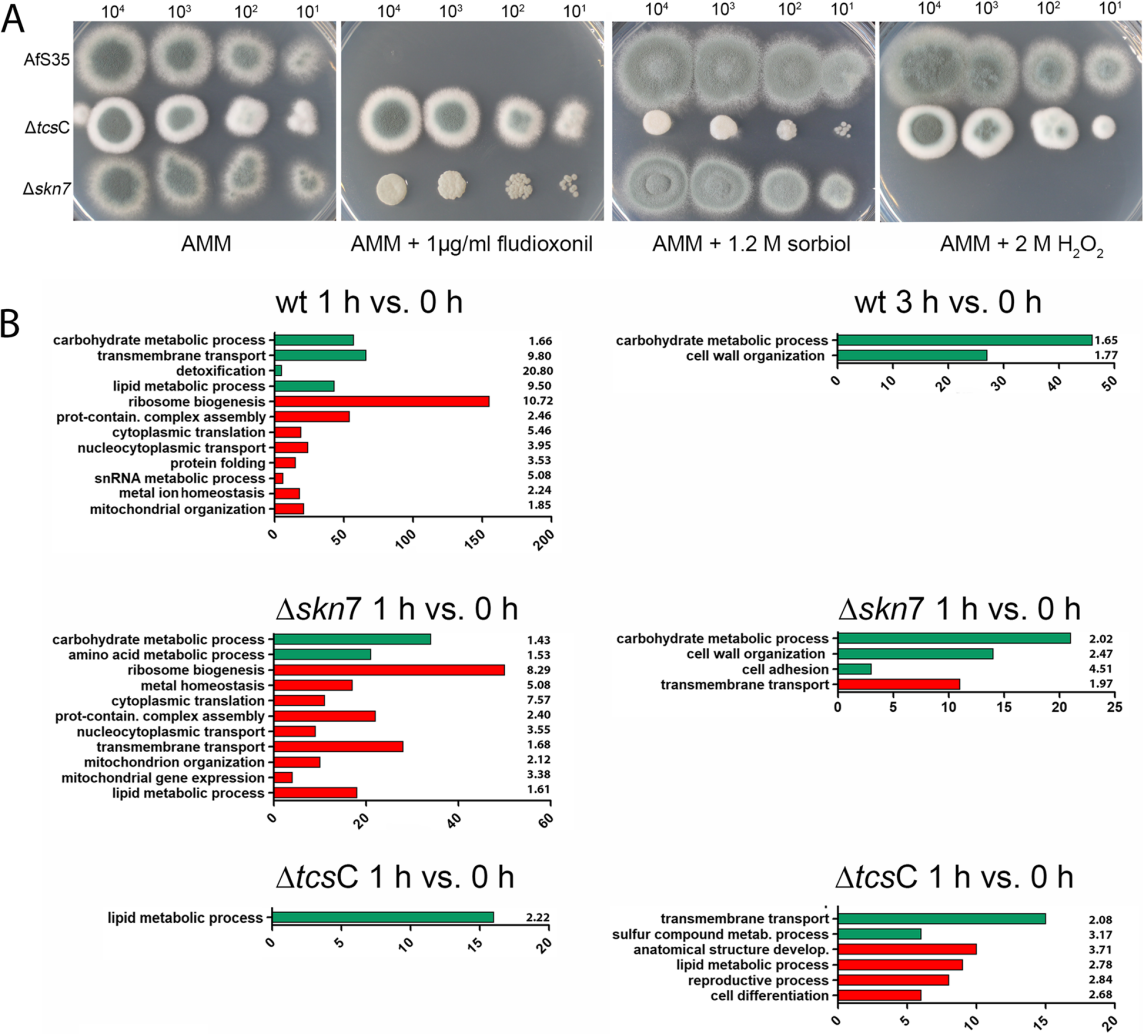
A phenotypic characterization of the AfS35 wild type, the Δ*skn*7 mutant and the Δ*tcs*C mutant is shown in **A** The indicated numbers of conidia were spotted onto AMM plates containing the indicated supplements. The images were taken after 48 h incubation at 37 °C, only the image of the sorbitol-containing plate was taken after 72 h. **B** Shows the enrichment of certain GO terms in the fludioxonil-induced transcriptional responses of the three strains. GO slim was used to identify enriched GO terms of the ontology “Biological Process”. Up-regulated genes are shown in green and down-regulated genes in red. The number of genes of the respective categories and the fold-enrichment values are indicated

The conidia of the three strains that were used for the transcriptional analysis were also analysed for their phenotypes on plates containing either 1 µg/ml fludioxonil, 1.2 M sorbitol or 2 M H_2_O_2_. As expected from previous studies [5, 8], the wild type was highly sensitive to fludioxonil, the Δ*tcs*C mutant was completely resistant and the Δ*skn*7 showed an intermediate phenotype. Under hyperosmotic stress, only the Δ*tcs*C mutant was impaired in growth, whereas the Δ*skn*7 was the only strain that showed a higher sensitivity to H_2_O_2_ (Fig. 2A).

In average, about 80% of the differentially expressed genes (DEGs) per comparison are supported by all four DEG tools**.** As mentioned in the methods section, the final DEG list is determined by deriving the intersection of DEGs reported by all four tools. In the further analysis, we focused on DEGs with absolute log_2_ fold change (log_2_FC) values greater than or equal 1.5. Using GO Slim, we searched for enriched GO Biological Processes in the three strains at both time points. These data are summarized in Fig. 2B. Up-regulation of genes belonging to the category Carbohydrate Metabolic Process is evident for the wild type and to a more moderate extent for the Δ*skn*7 mutant. A further analysis using the GO category Cellular Component revealed that the wild type is enriched for up-regulated cell wall-related genes.

Both strains show a similar down-regulation of genes belonging to the categories Ribosome Biogenesis, Cytoplasmic Translation, Mitochondrial Organization, Protein-containing Complex Assembly, Nucleocytoplasmic Transport and Metal Ion Homeostasis. Three-times more genes of the category Ribosome Biogenesis were down-regulated in the wild type compared to the Δ*skn*7 mutant (Fig. 2B). The category Lipid Metabolic Process showed an inverse regulation in the wild type and the Δ*skn*7 mutant and this included several genes involved in sterol biosynthesis that were up-regulated in the wild type, but down-regulated in the Δ*skn*7 mutant.

In a control experiment, we analysed five genes by qPCR that according to the RNA-seq data were differentially expressed in the wild type. Four genes of these genes were up- and one was down-regulated. The qPCR data confirmed this regulation; we obtained the following 2^-^^(ΔΔCt)^ values: 1.81 ± 0.29 for Afu6g06340, 0.77 ± 0.07 for Afu1g03352, 4.00 ± 1.82 for Afu1g15440/*ags*3, 1.89 ± 0.24 for Afu7g01010/*adh*1 and 2.81 ± 0.58 for Afu8g05710/*stl*1.

### Comparison of fludioxonil-responsive genes in *A. fumigatus* and *A. nidulans*

The impact of fludioxonil on the gene expression in *Aspergillus* species has been the subject of two previous studies. Three *A. fumigatus* genes were found to be highly up-regulated in response to fludioxonil, namely *cat*A, *dpr*A and *dpr*B [3]. In our data set, *cat*A (Afu6g03890) was up-regulated in the wild type (log_2_FC values: 1.92) and even stronger and more persistent in the Δ*skn*7 mutant (log_2_FC values: 4.10 at 1 h and 1.55 at 3 h). A similar strong and fludioxonil-induced up-regulation of *cat*A was observed for an *A. nidulans* Δ*srr*A mutant [7]; SrrA and Skn7 are orthologous proteins and share 71% identical residues. CatA is a cytosolic catalase in resting conidia that is hardly expressed in hyphae [12]. The *A. fumigatus* genes *drp*A (Afu4g00860) and *drp*B (Afu6g12180) are controlled by SakA and encode dehydrin-like proteins that protect cells against oxidative, osmotic and pH stress [13]. After 1 h in the presence of fludioxonil, both genes were up-regulated in the wild type and the Δ*skn*7 mutant, but only *drp*A showed a sustained up-regulation in both strains. From these three genes, only *drp*B reached a strong expression level (Additional Table 1). The more recently identified *drp*C gene [14] showed no response to fludioxonil.

Using DNA microarrays, Hagiwara et al. analysed the transcriptional response of *A. nidulans* 15 min after addition of fludioxonil [15]. Only genes that were differentially regulated by a factor of greater than 3 were analysed (this corresponds to a log_2_FC value of approximately 1.6), which led to the identification of 283 up- and 121 down-regulated genes. We searched for *A. fumigatus* orthologs for the 20 most up- and the 20 most down-regulated *A. nidulans* genes that were described in [15]. We identified *A. fumigatus* orthologs for 37 of these 40 genes and analysed them for differential expression in our data set. Six out of 17 showed a similar up- and nine out of 20 a similar down-regulation (Additional Table 1).

The most striking difference between the two data sets is that in *A. nidulans* only few DEGs were regulated in a SrrA-dependent manner [15], whereas several hundred DEGs in *A. fumigatus* were expressed in a Skn7-dependent manner. This is in line with previous data indicating a different importance of Skn7 and SrrA in the antifungal activity of fludioxonil [7, 8].

A common trend in the data sets obtained for *A. nidulans* and *A. fumigatus* is that fludioxonil triggers an up-regulation of genes encoding putative transporters and efflux pumps, whereas genes linked to ribosome biogenesis and translation are down-regulated. It is remarkable that for *A. fumigatus* this down-regulation of ribosomal genes was only transient; it was detectable after 1 h, but not after 3 h in the presence of fludioxonil.

### Comparison of the transcriptional responses of *A. fumigatus* to fludioxonil and hyperosmotic stress

**Fig. 3:**
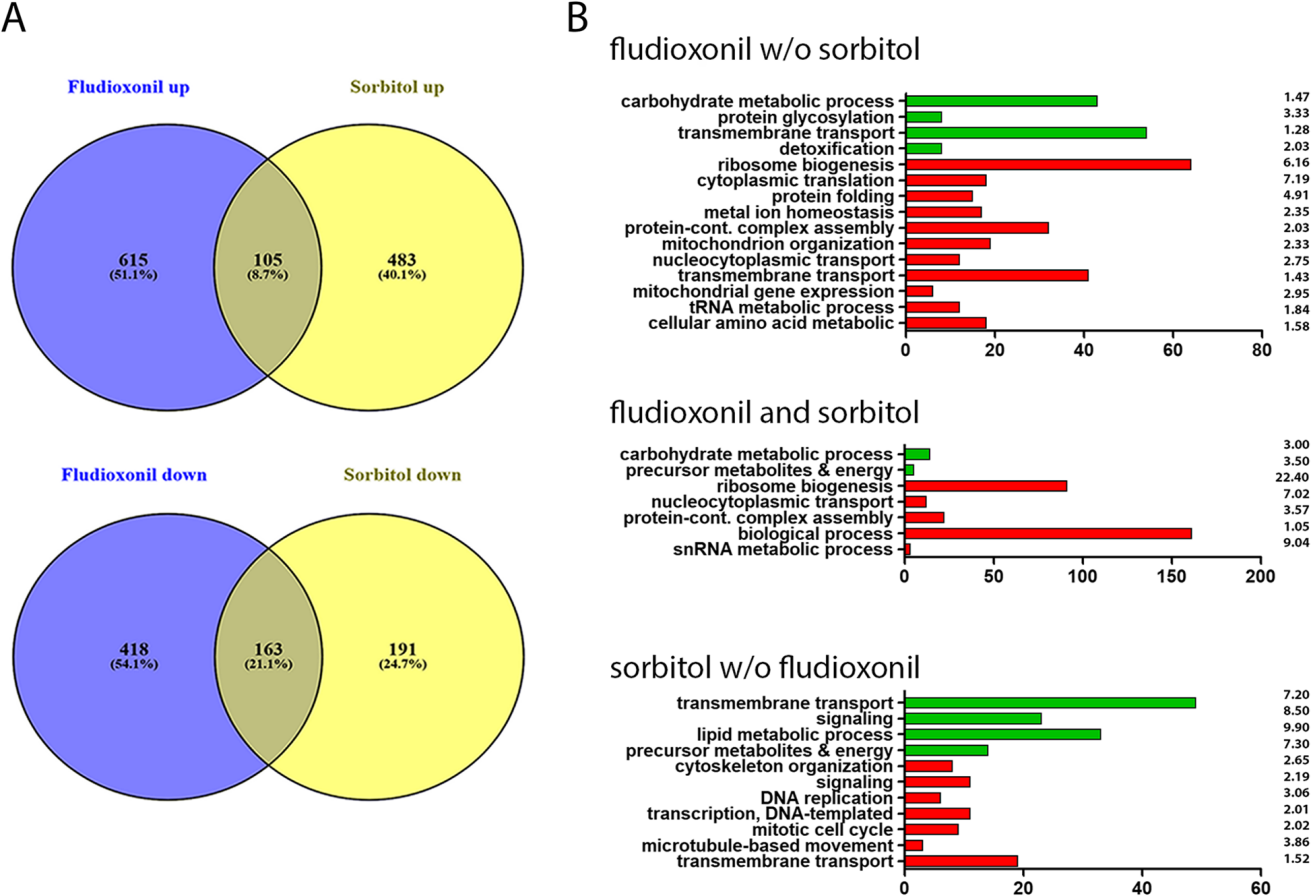
Comparison of the transcriptional responses of the *A. fumigatus* wild type to fludioxonil and hyperosmotic stress. The fludioxonil data were obtained after 1 h exposure of the wild type. The data on the hyperosmotic stress response were obtained from Pereira Silva et al*.* (2016) 10 min after exposure to 1 M sorbitol. A threshold value of 1.5 log_2_FC was applied to both data sets. **A** shows Venn diagrams of the up- und down-regulated genes in both responses. The enriched GO terms of the differentially regulated genes are summarized in **B**. The number of genes of the respective categories and the fold-enrichment values are indicated

Pereira Silva et al. have previously analysed the differential gene expression of *A. fumigatus* 10 min after a transfer to 1 M sorbitol-containing medium [16]. We compared these data to those obtained for the *A. fumigatus* wild type 1 h after addition of fludioxonil. The Venn diagrams presented in Fig. 3A show that both responses are clearly distinct, but nevertheless share a common core of 105 up- and 163-down-regulated genes. A search for enriched GO terms in these data was performed and the results are summarized in Fig. 3B. The jointly regulated genes comprise 14 up-regulated genes involved in carbohydrate metabolic processes, whereas the jointly down-regulated genes include many genes that are assigned to ribosome biogenesis and function.

The elimination of sorbitol-regulated genes from the DEGs identified after fludioxonil treatment (‘fludioxonil w/o sorbitol’) had only a minor impact on the enriched GO terms (compare Fig. 2 to Fig. 3B). This indicates that fludioxonil triggers a transcriptional response that is clearly distinct from the one induced by sorbitol. The GO terms that are enriched in the ‘sorbitol w/o fludioxonil’ group comprise up-regulated genes belonging to the categories Lipid Metabolic Processes and Signaling as well as down-regulated genes that are implicated in DNA replication.

### Comparison of the gene expression pattern of the three strains without fludioxonil treatment

Comparison of the transcriptional profiles of the control samples without fludioxonil treatment (t = 0 h) revealed a high level of similarity (Fig. 1), but also some differences. Compared to the wild type, the Δ*skn*7 mutant had 29 up- and 17 down-regulated genes and the Δ*tcs*C mutant had 52 up- and 15 down-regulated genes. In both mutants, an equally orientated regulation was evident for 12 up- and one down-regulated genes compared to wild type. These genes are indicated in bold in Additional Table 2. The largest number of DEGs was found for ∆*skn*7 versus ∆*tcs*C with 110 genes (53 up- and 57 down-regulated in the ∆*skn*7 mutant) (Additional Table 2).

In the ∆*skn*7 mutant, expression of the mycelial catalase 1 (Afu3g02270) was weaker than in the other strains (log_2_FC of -1.66 and -1.44), which correlates well to the previously described higher sensitivity of the ∆*skn*7 mutant to H_2_O_2_ [8, 17]. In this context, it is notable that fludioxonil induced a marked up-regulation of the *cat*1 gene, but only in the ∆*skn*7 mutant (log_2_FC of 2.33 after 1 h and 3.10 after 3 h). After 3 h, the expression level of *cat*1 in the ∆*skn*7 mutant was two- to four-fold higher than in the other strains.

The sensor kinase *tcs*B (Afu2g00660), an ortholog of the *S. cerevisiae* osmosensor Sln1p, was up-regulated in the ∆*tcs*C mutant compared to ∆*skn*7 (log_2_FC value: 1.63) and to a lesser extend also compared to the wild type (log_2_FC value: 1.04).

Three adjacent genes Afu2g17820, Afu2g17830 and Afu2g17840 were down-regulated in the ∆*tcs*C mutant compared to the other strains (log_2_FC values range from -1.94 to -2.43).

### Comparison of the fludioxonil-induced differential gene expression in the three strains

**Fig. 4:**
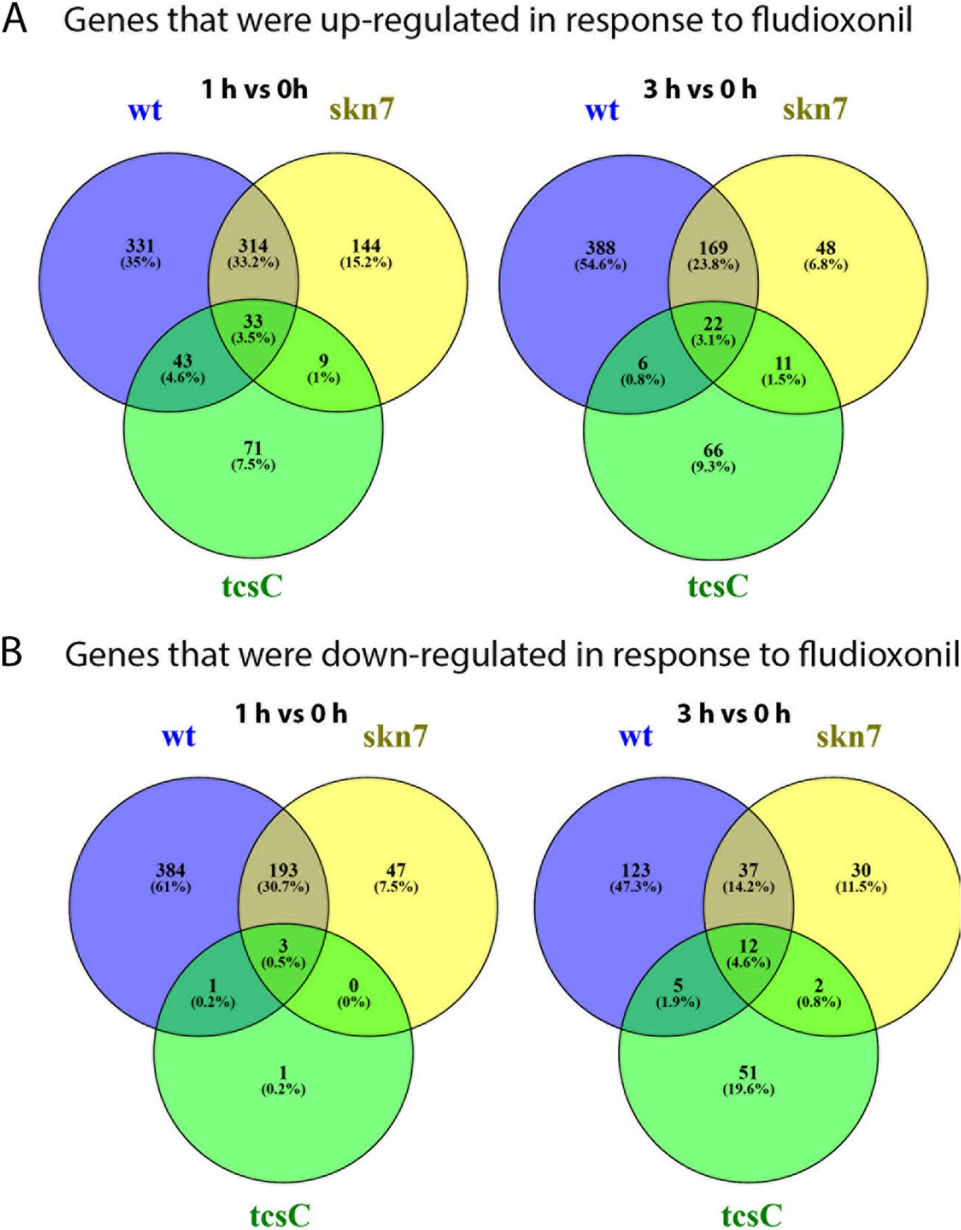
Venn diagrams showing differentially expressed genes of the three strains in response to fludioxonil (threshold value: 1.5 log_2_FC). *wt* Wild type strain AfS35, *tcsC* Δ*tcs*C and *skn7* Δ*skn*7 mutant

The Δ*tcs*C mutant showed by far the weakest transcriptional response to fludioxonil. Only 161 genes were differentially expressed after 1 h of exposure (156 up- and 5 down-regulated) and 175 DEGs were found after 3 h (105 up- and 70 down-regulated). In the wild type, 1301 DEGs were identified after 1 h (720 up- and 581 down-regulated) and 762 DEGs after 3 h (585 up- and 177 down-regulated) (Fig. 4). For the ∆*skn*7 mutant, we found an intermediate response with 743 DEGs at 1 h (500 up- and 243 down-regulated) and 331 DEGs at 3 h (250 up- and 81 down-regulated) (Fig. 4). Hence, the number of DEGs of the three strains correlates well with their sensitivity to fludioxonil, being highest for the wild type and lowest for the ∆*tcs*C mutant.

### Identification of a small set of fludioxonil-responsive genes found in all three strains

**Fig. 5:**
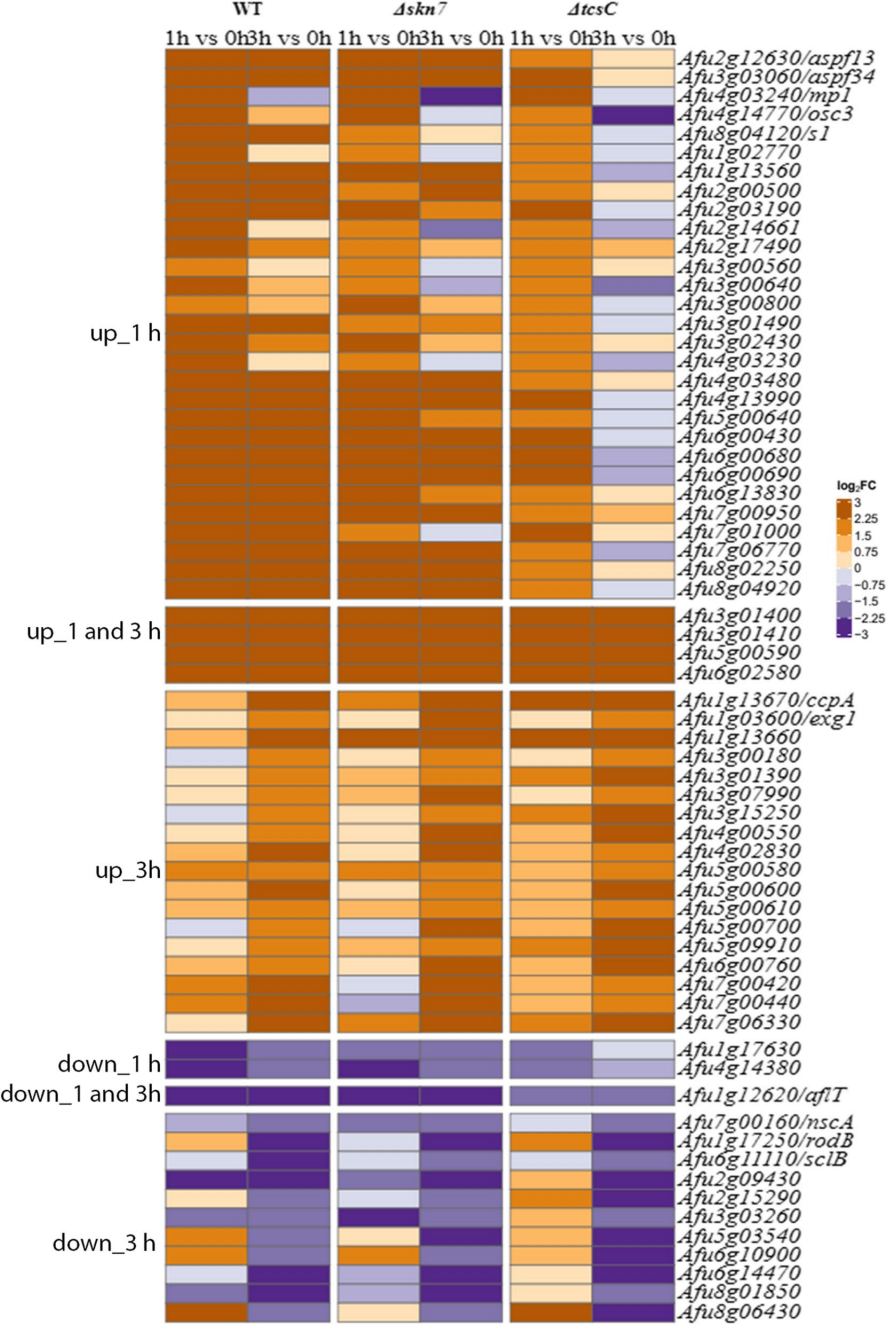
Heat maps of genes that were differentially regulated in all three strains. Genes that were up-regulated in all strains after 1 h, at both time points and after 3 h are listed in upper three blocks. Genes that were down-regulated in all strains after 1 h, both time points and 3 h are listed in the lower three blocks. The colour code of the heat map is given at the right margin

Only few genes showed a similar response in all three strains; heat maps of these genes are shown in Fig. 5. Only four genes were up-regulated at both time points. Two of them are the neighbouring genes Afu3g01400 and Afu3g01410 that encode a putative ABC multidrug transporter and a putative polyketide synthase. Afu3g01400 showed not only a marked up-regulation, but was also a gene with a particularly strong expression level in all three strains. Afu3g01400 and Afu3g01410 belong to a putative secondary metabolite gene cluster with Afu3g01410 as its backbone enzyme [18]. Four other genes that belong to this putative gene cluster, namely Afu3g01450, Afu3g01480, Afu3g01490 and Afu3g01500, were also up-regulated, but only in the wild type and the Δ*skn*7 mutant.

It is remarkable that the three genes with the strongest up-regulation in response to fludioxonil in the wild type are among those being overexpressed in all three strains; these genes were already strongly expressed at 0 h and encode an IgE-binding protein (Afu6g00430), an extracellular protein of unknown function (Afu6g00690) and the putative cell wall-protein PhiA (Afu3g03060). The up-regulation of these genes was most prominent in the wild type (log_2_FC values > 9), intermediate in the ∆*skn*7 mutant (log_2_FC values between 5 and 7) and weakest in the ∆*tcs*C mutant (log_2_FC values between 2 and 3). A comparison of the DEGs revealed only 12 fludioxonil up-regulated genes (FUGs) and one fludioxonil down-regulated gene (FDG) showing a consistent regulation at both time points (Fig. 5). None of the DEGs that were regulated in all strains after 1 h showed a similar response after exposure to 1 M sorbitol [16] indicating that this part of the response represents a fludioxonil-specific signature.

### Genes that are exclusively regulated in the *A. fumigatus* wild type

Genes that showed a wild type-specific regulation may be helpful to gain further insights in the impact of fludioxonil on the fungal physiology. We found 330 and 388 wild type-specific FUGs after 1 h and 3 h as well as 384 and 123 wild type-specific FDGs after 1 h and 3 h. Of these genes, 100 were up- and 30 down-regulated at both time points (Additional Table 3).

**Fig. 6:**
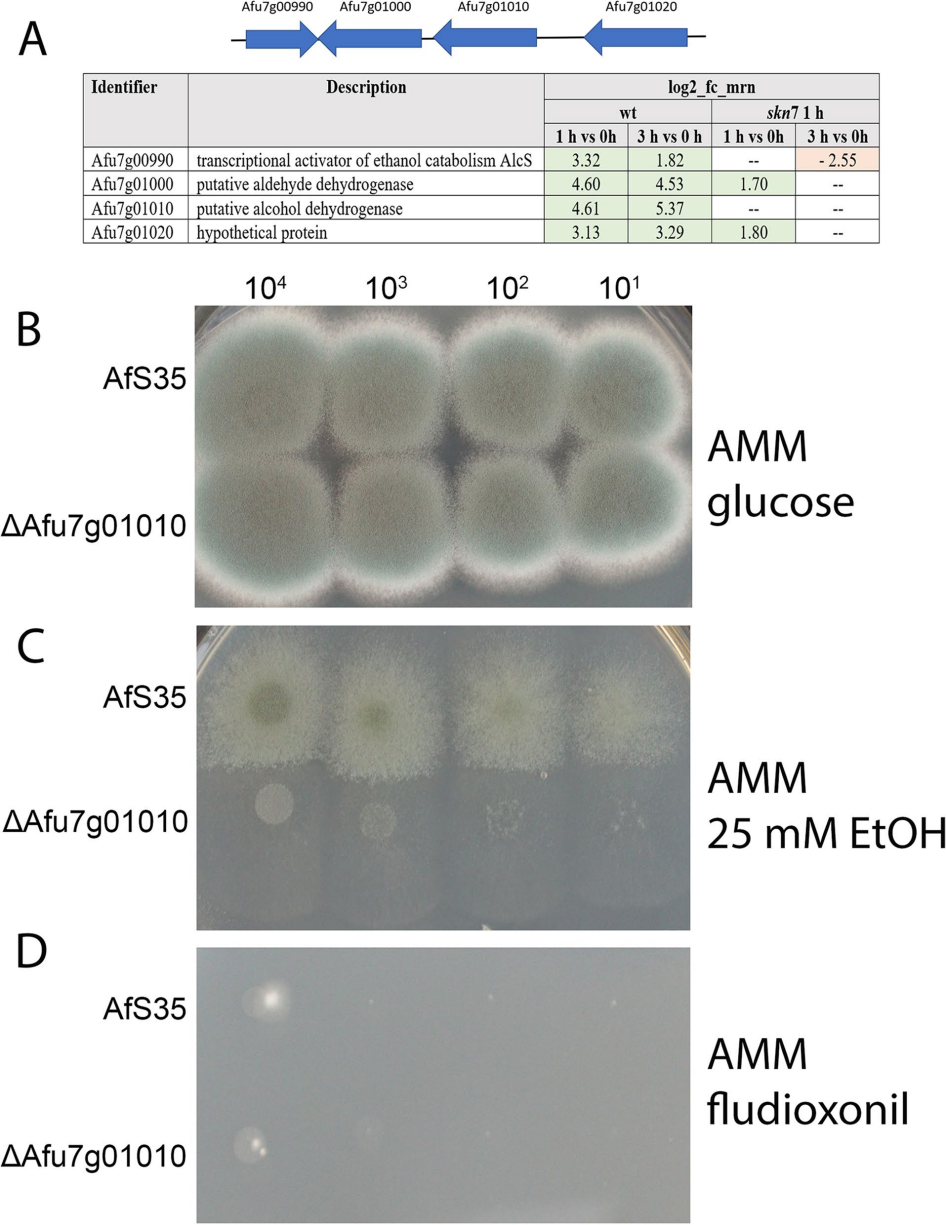
Fludioxonil-induced regulation of genes involved in the catabolism of alcohol. **A** The organization of the *A. fumigatus alc* gene cluster is schematically depicted and the differential expression is indicated as log_2_-fc.mrn values. The deletion mutant of the putative alcohol dehydrogenase Afu7g01010 showed a normal growth on AMM (**B**), a severe growth defect on AMM containing 25 mM ethanol as sole carbon source (**C**) and was as sensitive to fludioxonil (1 µg/ml) as the parental strain AfS35 (**D**). The number of spores per spot is indicated

The two neighbouring genes Afu7g01000 and Afu7g01010 encode a putative alcohol and a putative acetaldehyde dehydrogenase and are both strongly up-regulated at both time points (Fig. 6A). The two flanking genes Afu7g0990 and Afu7g01020 that encode a putative membrane protein and a small protein of unknown function, were also up-regulated at both time points (Fig. 6A). The four *A. nidulans* orthologs of these genes constitute the so-called *alc* gene cluster [19]. None of the four *A. fumigatus* genes is regulated in response to sorbitol [16].

Afu7g01010 is an ortholog of *Candida albicans* Adh1, which catalyses the oxidation of methylglyoxal to pyruvate [20]. This is a particularly interesting gene, since Brandhorst et al. [21] showed that in *Blastomyces dermatitidis*, methylglyoxal plays a crucial role in the antifungal activity of fludioxonil. The up-regulation of the triosephosphate isomerase gene (Afu2g11020) in the *A. fumigatus* wild type could also be relevant in this context (log_2_FC values of 1.42 at 1 h and 2.12 at 3 h). We hypothesized that Adh1 could be involved in the detoxification of methylglyoxal in *A. fumigatus* and therefore deleted this gene. The mutant grew normally on Aspergillus Minimal Medium (AMM) (Fig. 6B), showed a severe growth defect on plates containing ethanol as sole carbon source (Fig. 6C) and was as sensitive to fludioxonil as the parental strain AfS35 (Fig. 6D). These findings verify that Afu7g01010 encodes an ethanol dehydrogenase, but rule out that this gene is essential for the antifungal activity of fludioxonil. Brandhorst et al. [21] hypothesized that in *B. dermatididis* methylglyoxal is degraded by the lactoylglutathione lyase Glo1. Two potential orthologs of Glo1 exist in *A. fumigatus*, namely Afu6g07940 and Afu2g13550. These genes are either not or only very weakly up-regulated in the wild type. We furthermore tested whether the *A. fumigatus* wild type and the Δ*tcs*C mutant differ in their sensitivity to methylglyoxal, but observed no differences in drop dilution experiments (data not shown).

Afu6g10650 and Afu6g10660 are two neighbouring genes that are strongly down-regulated, in a wild type-specific manner (log_2_FC values: -2.42 after 1 h and -2.36 after 3 h as well as -2.43 after 1 h and -2.40 after 3 h, respectively); these genes encode the subunits of the cytosolic ATP citrate lyase. The malate dehydrogenase (Afu7g02420), the second enzyme of the citrate-malate-shuttle is also down-regulated, but only after 1 h (log_2_FC value: -2.15). Concomitant, we observed an up-regulation of genes encoding components of the glyoxylate cycle: the isocitrate lyase AcuD (Afu4g13510; log_2_FC values: 3.50 at 1 h and 3.43 at 3 h), the malate synthase (Afu6g03540; log_2_FC value: 1.58 at 1 h) and the citrate synthase (Afu6g03590; log_2_FC values: 3.29 at 1 h and 2.22 at 3 h). This indicates a fludioxonil-induced and wild type-specific re-programming of the basic metabolism towards alternative carbon sources. It is remarkable that with the exception of Afu6g03590, all other genes show a similar regulation in response to hyperosmotic stress [16] suggesting that this metabolic reorganisation is important to increase the internal osmotic pressure. The wild type-specific response of these genes to fludioxonil suggests that this regulation depends on Skn7. This may provide an explanation for the importance of Skn7 for the hyperosmotic stress response in *A. fumigatus* [8].

### Genes that were exclusively regulated in the ∆*tcs*C or the Δ*skn*7 mutant

The ∆*tcs*C mutant is resistant to fludioxonil and shows only a limited transcriptional response to this agent. Using a threshold value of 1.5, we identified 71 ∆*tcs*C-specific FUGs after 1 h and 66 after 3 h; eight of these DEGs were up-regulated at both time points. A small number of ∆*tcs*C-specific DEGs were down-regulated (one at 1 h and 51 at 3 h). Hence, the ∆*tcs*C-specific transcriptional response to fludioxonil was limited, weak and not persisting. No enrichment of GO terms was evident for these genes.

For the Δ*skn*7 mutant, a specific up-regulation was found for 144 genes after 1 h and 48 genes after 3 h, whereas 47 and 30 genes were down-regulated in a ∆*skn*7-specific manner after 1 and 3 h, respectively. The GO terms Cell Wall (9 out of 209 genes) and Oxidoreductase Activity (23 out of 956) were enriched in the 1 h group of ∆*skn*7-specific up-regulated genes. In the ∆*skn*7-specific down-regulated genes, the 1 h group was weakly enriched for Structural Constituent of Ribosome (8 out of 143 genes) and the 3 h group for Extracellular Region (6 out of 374 genes). These data provide no obvious clues for a better understanding of the antifungal impact of fludioxonil; they suggest instead that this antifungal activity relies most likely on genes that are differentially expressed in a wild type-specific manner.

### Genes encoding proteins of the HOG pathway

TcsC (Afu2g03560) and most other genes encoding components of the HOG pathway showed no response to fludioxonil. Only the *ypd*1 gene (Afu4g10280) was up-regulated in the wild type strain after 1 h (log_2_FC value: 1.49) and in the *∆skn*7 mutant at both time points (log_2_FC value: 2.36 at 1 h and 1.75 at 3 h). The potentially HOG-linked gene *tcs*B (Afu2g00660) was down-regulated in the wild type (log_2_FC value: -1.75 at 1 h and -2.11 at 3 h). Afu3g11330 encodes the transcription factor AtfA, which operates downstream of SakA [16]. This gene showed no differential expression in response to fludioxonil. In the sorbitol data set, *ypd*1 and *tcs*B show a similar regulation as in the current study, whereas *atf*A and the genes encoding the HOG pathway proteins Pbs2 and SakA are strongly expressed in response to a high sorbitol concentration [16], but not in response to fludioxonil. The *atf*A ortholog in *A. nidulans* (AN2911) showed an elevated transcription in response to fludioxonil that was dependent on SskA and HogA [15].

### Glycerol metabolism

The characteristic fludioxonil-induced swelling was initially described as a consequence of an increased internal osmotic pressure [22], but for *A. fumigatus* recent data indicate that changes in the cell wall architecture are also important [8]. Glycerol is a compatible solute and its concentration can be substantially increased to counterbalance an elevated extracellular osmotic pressure without deleterious side effects for the cellular machinery. We have recently shown that the fludioxonil-induced increase of the internal glycerol concentration is largely, but not completely dependent on TcsC [8]. We therefore tried to identify DEGs with a potential impact on the cytoplasmic glycerol concentration.

Glycerol can be synthesized from dihydroxyacetone by a glycerol dehydrogenase, or alternatively from dihydroxyacetone phosphate via glycerol-3-phosphate (G-3-P). The *A. nidulans* G-3-P dehydrogenase GfdA (AN0351) generates G-3-P from dihydroxyacetone phosphate, but this gene is dispensable for the adaptive response to hyperosmotic stress in *A.* *nidulans* [23] and it is not regulated by fludioxonil [15]. The expression of the second *A. nidulans* G-3-P dehydrogenase gene *gfd*B is, in contrast, strongly up-regulated in response to fludioxonil [15], whereas the two putative *A. fumigatus* G-3-P dehydrogenase genes (Afu1g02150 and Afu2g08250) showed no differential expression. The glycerol dehydrogenase GldB (AN5563) plays an important role in the adaptive response of *A.* *nidulans* to hyperosmotic stress [24], but neither *gld*B nor its *A. fumigatus* ortholog Afu4g11730 showed a significant induction upon fludioxonil treatment. The mentioned glycerol-related *A. fumigatus* genes were also not regulated in response to hyperosmotic stress with only one exception: the putative G-3-P dehydrogenase gene Afu2g0820 is strongly up-regulated in this data set [16].

**Fig. 7:**
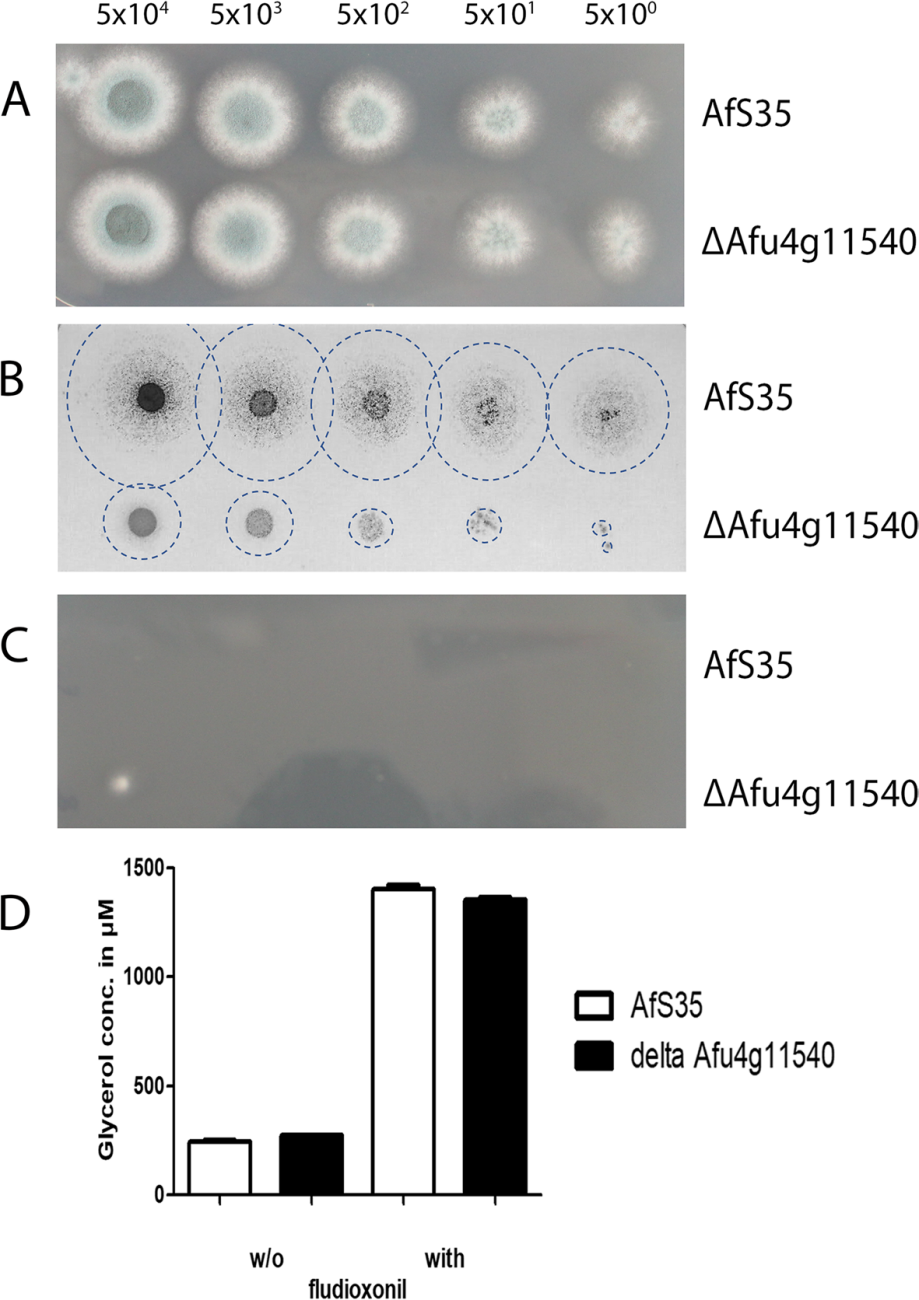
Characterization of a deletion mutant lacking Afu4g11540 that encodes a putative glycerol kinase. The deletion mutant and the parental strain AfS35 were compared in a drop dilution assay on AMM (**A**), AMM with glycerol (1 g/l) as sole carbon source (**B**) and AMM supplemented with 1 µg/ml fludioxonil (**C**). Colonies that grew on the glycerol-containing plates were difficult to visualize due to their thin and spread appearance (**B**); the edges of these colonies are therefore indicated by discontinuous circles. The number of spores per spot is indicated. **D** shows the glycerol concentrations of cellular extracts obtained either from hyphae after 6 h in the presence of fludioxonil (1 µg/ml) or from non-treated controls

Only few DEGs in our data set are possibly involved in the glycerol metabolism, but the genes encoding a putative glycerol dehydrogenase (Afu1g09930) and a putative glycerol kinase (Afu4g11540) were up-regulated in the wild type and the ∆*skn*7 mutant, but only after 1 h (Afu1g09930: log_2_FC values of 1.72 and 2.50, respectively; Afu4g11540: log_2_FC values of 2.12 and 2.49, respectively). Interestingly, both genes are also up-regulated in response to hyperosmotic stress [16]. Since both genes had not been characterized so far, we deleted Afu4g11540 in strain AfS35. The mutant grew normally on AMM (Fig. 7A), but was severely impaired in growth on plates containing glycerol as sole carbon source (Fig. 7B). This indicates that Afu4g11540 is important for the assimilation of glycerol, as previously reported for the homologous yeast glycerol kinase Gut1p [25]. The mutant lacking Afu4g11540 was as sensitive to fludioxonil as the wild type strain (Fig. 7C) and showed a wild type-like increase of its cytoplasmic glycerol concentration in response to fludioxonil (Fig. 7D), which demonstrates that this putative glycerol kinase is dispensable for the antifungal impact of fludioxonil.

### Regulation of putative secondary metabolite biosynthetic gene clusters

**Fig. 8:**
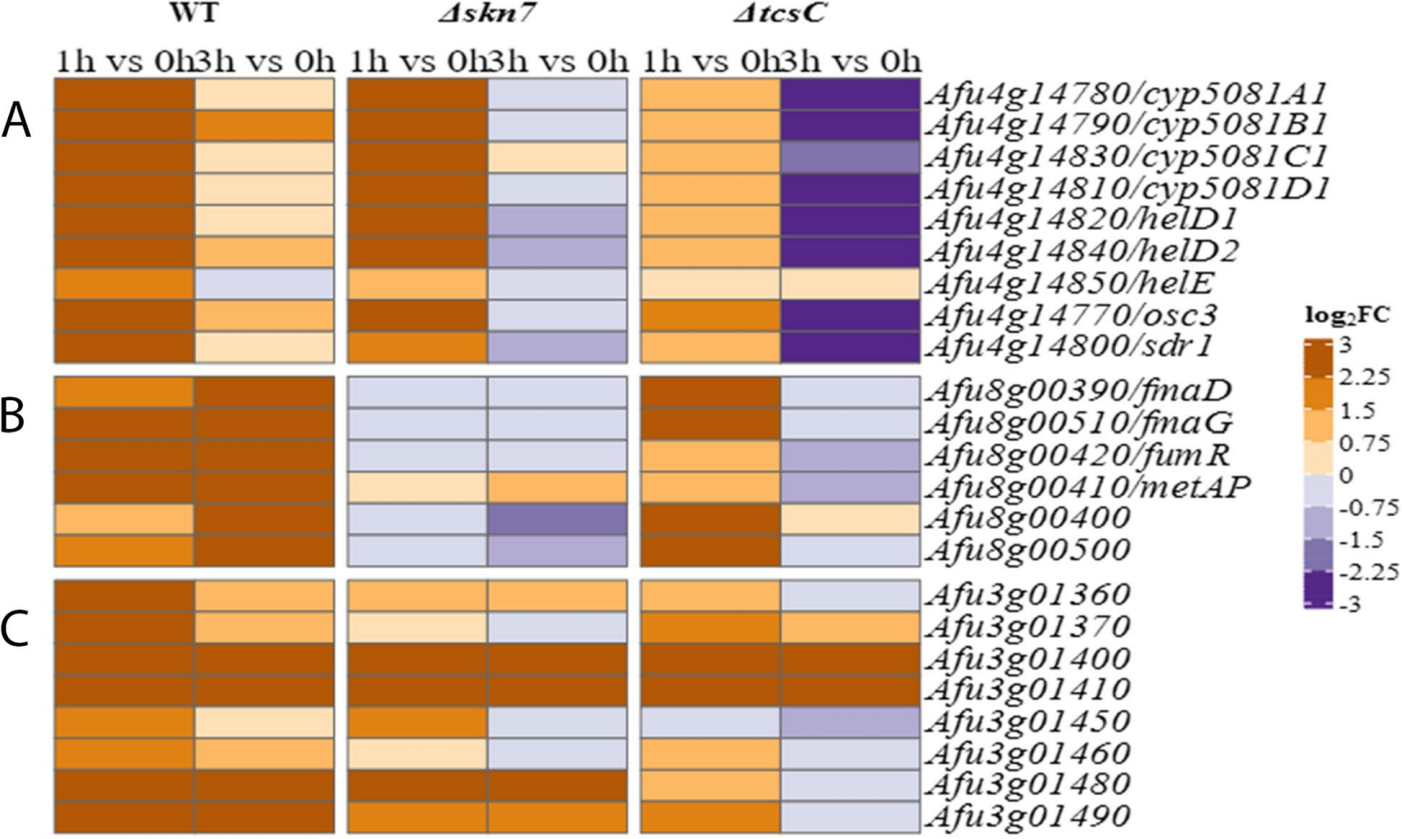
Heat maps of three *A. fumigatus* gene clusters that were differential expressed in the three strains in response to fludioxonil. These include genes that encode proteins for the biosynthesis of helvolic acid (**A**), fumagillin (**B**) and a yet unknown product (**C**). The colour code of the heat map is given at the right margin

The up-regulation of Afu3g01400 and Afu3g01410, two genes belonging to a putative secondary metabolite biosynthetic gene cluster has been mentioned above. In addition, we observed an up-regulation of genes involved in the biosynthesis of the antibacterial nortriterpenoid helvolic acid (Afu4g14770—Afu4g14850), both in the wild type and the Δ*skn*7 mutant, but only after 1 h of fludioxonil treatment (Fig. 8A). Moreover, we found that six out of 13 genes of the fumagillin gene cluster were up-regulated, in a wild type-specific manner and this differential expression was particularly strong after 3 h (Fig. 8B). Another set of clustered genes that was up-regulated in the wild type and the Δ*skn*7 mutant belongs to a yet uncharacterized, highly reduced polyketide gene cluster listed in [26] (Fig. 8C). Hence, the fludioxonil-induced stress leads to an elevated transcription of three gene clusters that are implicated in the biosynthesis of different secondary metabolites. Remarkably, none of these genes is regulated in response to hyperosmotic stress [16].

### Iron uptake and oxidative stress

**Fig. 9:**
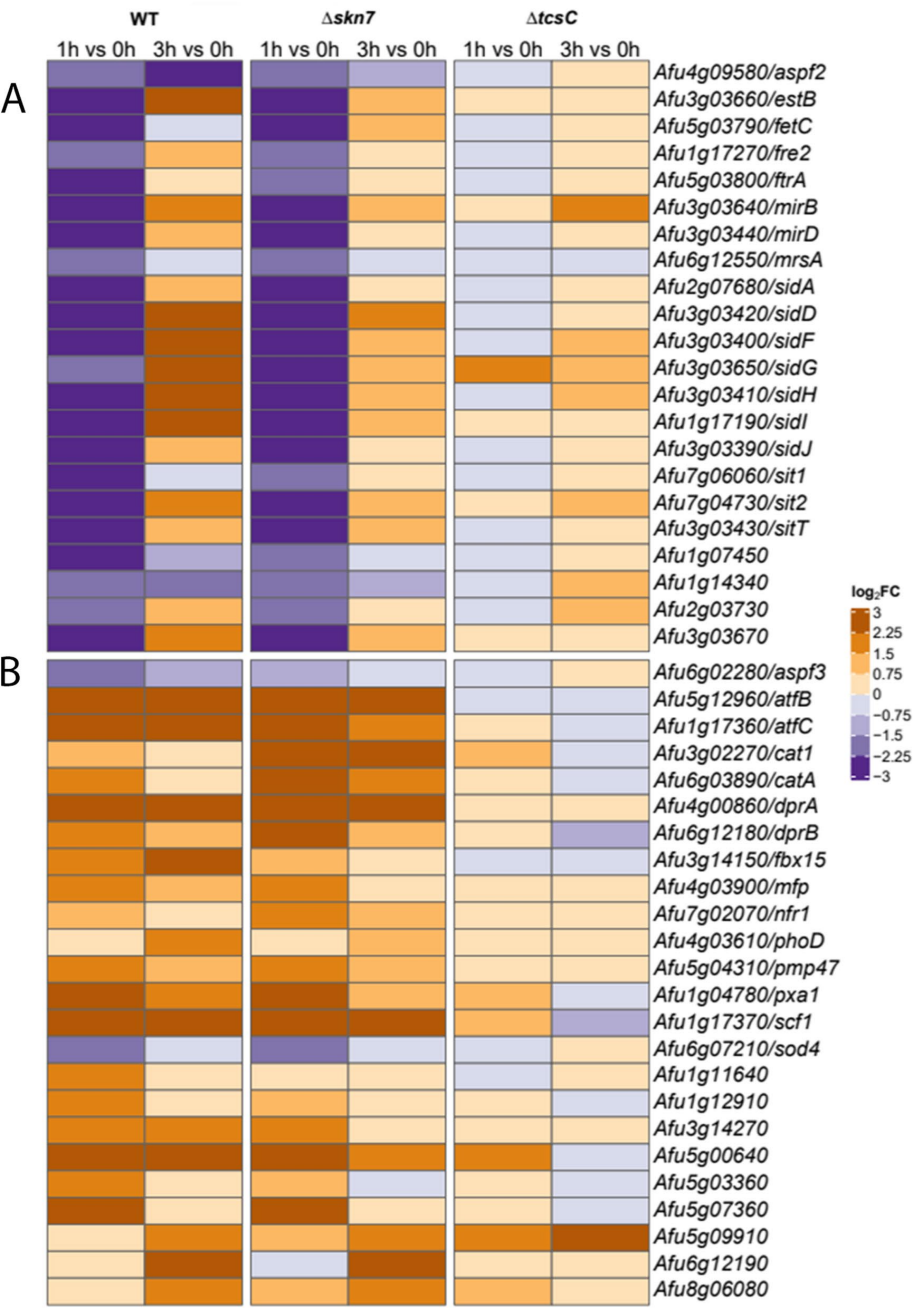
Genes that are implicated in iron homeostasis, oxidative stress and peroxisome function showed a transcriptional response to fludioxonil. DEGs that are involved in iron and metal ion homeostasis are summarized in **A**. DEGs encoding proteins involved in the response to oxidative stress or of peroxisomal origin are listed in **B**. The colour code of the heat map is given at the right margin

N',N'',N'''-triacetylfusarinine C (TAFC) is an important extracellular siderophore and crucial for the virulence of *A. fumigatus* [27]. After 1 h, the genes that are required for the synthesis of TAFC are down-regulated in the wild type and the Δ*skn*7 mutant. In the former, these genes are strongly up-regulated after 3 h, whereas the expression levels in the Δ*skn*7 mutant were only slightly elevated after 3 h (Fig. 9A). A similar transcriptional pattern was found for the two major facilitator-type transporters MirB and MirD that mediate the uptake of TAFC-chelated iron [27], and the same applies for several other genes implicated in siderophore biosynthesis (Fig. 9A). This regulation does not occur in response to hyperosmotic stress [16] and therefore appears to be a characteristic element of the fludioxonil stress response.

Several genes implicated in the response to oxidative stress are also up-regulated in the wild type and the Δ*skn*7 mutant (Fig. 9B). The absence of this transcriptional response in the Δ*tcs*C mutant suggests that fludioxonil-derived signals are processed via TcsC to trigger a certain level of oxidative stress. Peroxisomes are cellular organelles that play an important role in the oxidative metabolism and also contribute to the protective response to oxidative stress [28]. As shown in Fig. 9B, we observed a fludioxonil-induced up-regulation of several peroxisomal and oxidative stress-related genes in the wild type and the Δ*skn*7 mutant. After exposure to 1 M sorbitol, nine of these 24 genes were also up-regulated including those of CatA and the dehydrin-like proteins DrpA and DrpB [16].

### Cell wall-related genes

The characteristic ballooning of fludioxonil-treated fungal cells indicates that the cells are unable to maintain their normal cellular size due to an instability of their cell wall. The driving force behind this process is an osmotic gradient over the cytoplasmic membrane that is characteristic for fludioxonil-treated cells. The combination of both processes entails a massive influx of water and the characteristic swelling, a feature that does not occur in response to hyperosmotic stress.

**Fig. 10:**
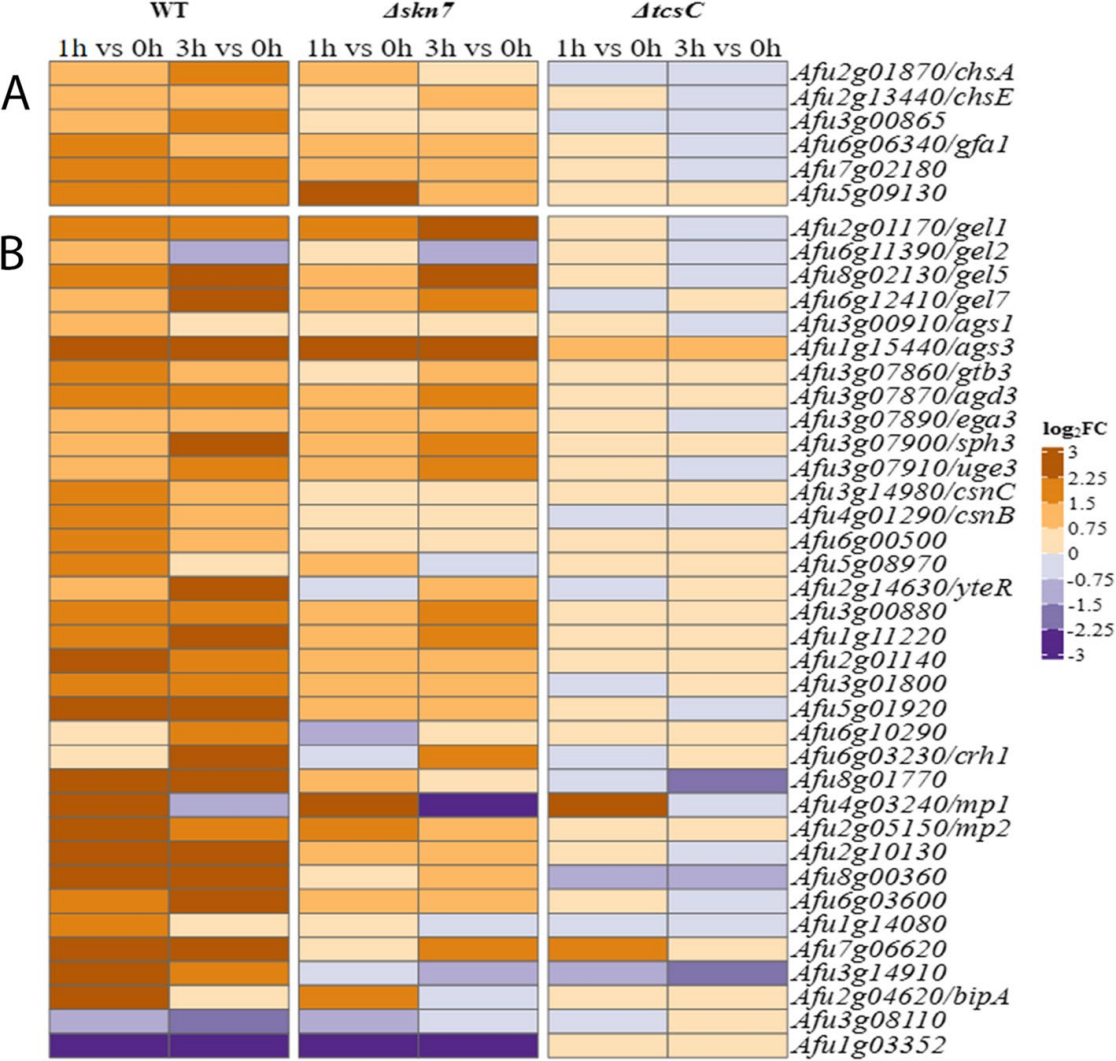
Heat maps of cell wall-related genes of the three strains that were differentially expressed in response to fludioxonil. Genes that are implicated in the biosynthesis of chitin are listed in **A**. Other differentially expressed genes that encode cell wall-related proteins are summarized in **B**. The colour code of the heat map is given at the right margin

We have previously shown that treatment with fludioxonil results in a shedding of the cell wall components galactomannan and β-1,3-glucan, and an increased binding of the chitin-specific dye Calcofluor white [6, 29]. The RNA-seq data indicate that many known or putative cell wall-associated genes are up-regulated in a wild type-specific manner (Fig. 10), which includes genes that are involved in the biosynthesis of chitin (Fig. 10A). The glucosamine-fructose-6-phosphate aminotransferase (Afu6g06340) is an already strongly expressed gene under steady state conditions that is further up-regulated in the wild type in response to fludioxonil. Another result that fits well to an increase of the chitin content is the sustained and wild type-specific up-regulation of Afu7g02180, a gene encoding an UDP-N-acetylglucosamine pyrophosphorylase that produces the chitin precursor UDP-N-acetylglucosamine. Chitosan is a deacetylated form of chitin and three putative chitosanases Afu4g01290 (CsnB), Afu3g14980 (CsnC) and Afu6g00500 show a wild type-specific up-regulation by fludioxonil after 1 h (Fig. 10B). None of these nine chitin-related genes is regulated in response to 1 M sorbitol [16].

From the three α-1,3-glucan synthase genes of *A. fumigatus* [30, 31], only *ags*1 (Afu3g00910) and *ags*3 (Afu1g15440) are strongly expressed. The weakly expressed *ags*2 gene (Afu2g11270) showed no response to fludioxonil. For *ags*1, the already high expression level under ambient conditions was further increased in the wild type after 1 h (log_2_FC value: 1.43). Expression of *ags*3 showed a strong and persistent up-regulation in both, the wild type and the Δ*skn*7 mutant (Fig. 10B). Afu1g03352 encodes a putative α-1,3-glucanase and is one of the most strongly down-regulated genes 1 and 3 h after addition of fludioxonil, both in the wild type and the Δ*skn*7 mutant (Fig. 10B). Hence, the α-1,3-glucan synthases genes *ags*1 and *ags*3 and the putative α-1,3-glucanase gene Afu1g03352 are inversely regulated in response to fludioxonil.

From the family of β-1,3-glucanosyltransferases, three genes (*gel*1, *gel*5 and *gel*7) showed a strong, fludioxonil-induced up-regulation. *gel*1 (Afu2g01170) was a FUG in the wild type and the Δ*skn*7 mutant, whereas the up-regulation of *gel*5 (Afu8g02130) and *gel*7 (Afu6g12410) was more pronounced in the wild type (Fig. 10B). The putative β-1,3-glucanosyltransferase gene *gel*2 (Afu6g11390) was not regulated in response to fludioxonil (Fig. 10B).

Galactosaminogalactan (GAG) is an exopolysaccharide and a major virulence determinant of *A. fumigatus* [32]. A cluster of five genes is responsible for GAG biosynthesis [33] and these genes were up-regulated in response to fludioxonil, both in the wild type and the Δ*skn*7 mutant (Fig. 10B).

The cell wall galactomannoprotein MP1 (Afu4g03240) was strongly up-regulated in the wild type and the Δ*skn*7 mutant, but only after 1 h. The cell wall galactomannoprotein MP2 (Afu2g05150) was also much stronger up-regulated in the wild type than in the Δ*skn*7 mutant (Fig. 10B). Many other cell wall-associated and putative GPI-anchored proteins were also up-regulated and this response was largely restricted to the wild type (Fig. 10B) indicating that fludioxonil triggers substantial and strain-specific changes in the architecture of the cellular envelope.

Only two of these 35 cell wall-related genes were up-regulated in response to hyperosmotic stress: the GAG biosynthesis genes *ega*3 (Afu3g07890) and *uge*3 (Afu3g07910) [16]. This underlines that cell wall rearrangements are a defining feature of the antifungal impact of fludioxonil.

### Fludioxonil-induced changes in the composition of the cell wall

**Fig. 11:**
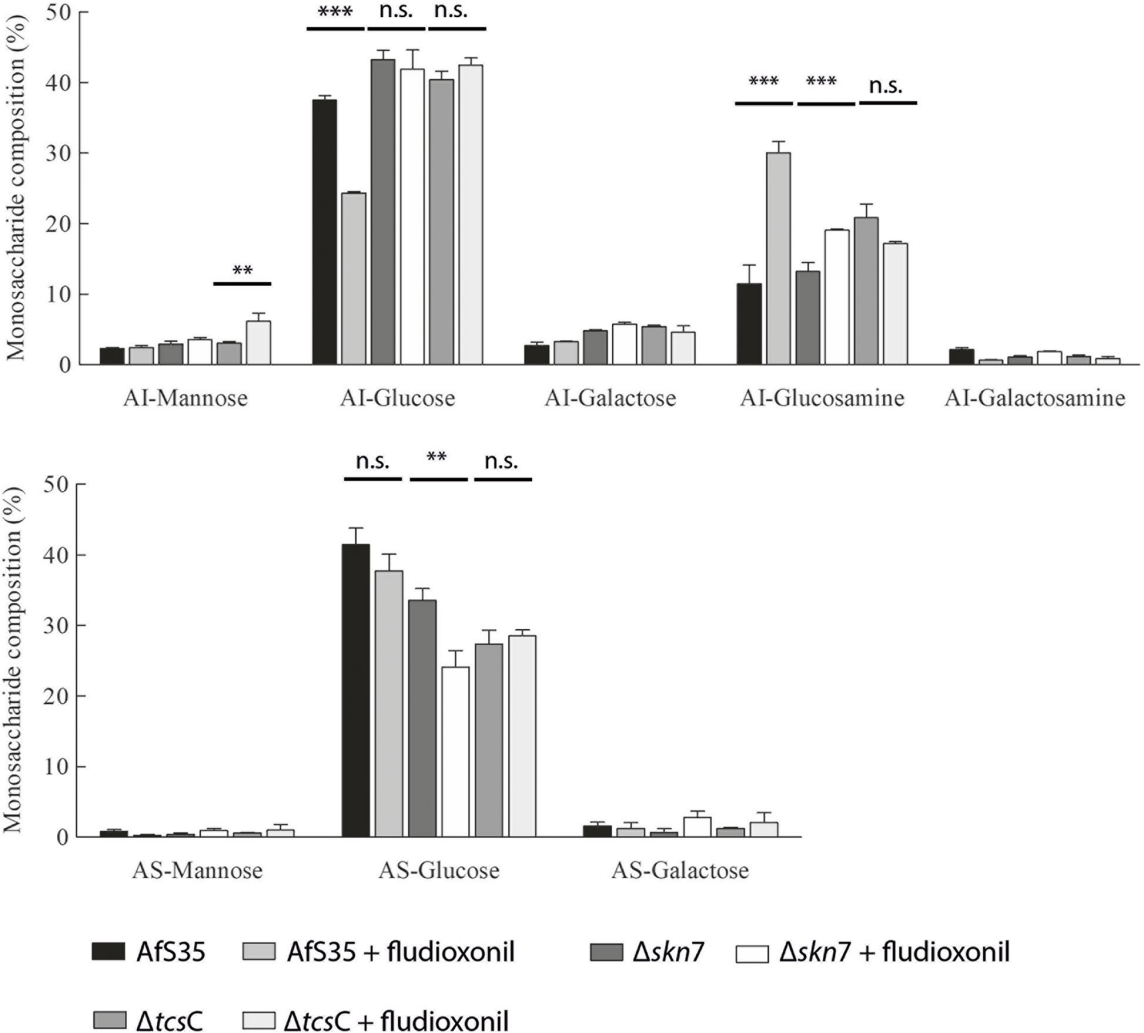
Cell wall analysis of the three strains grown in the presence and absence of fludioxonil. *AI* Alkali-insoluble fraction, *AS* Alkali-soluble fraction, *** *p* < 0.001, ** *p* < 0.01, *n.s*. Not significant

In parallel to the transcriptional study, we also analysed the cell wall composition of the three strains before and after fludioxonil treatment. Fludioxonil caused a strong increase of the glucosamine content in the alkali-insoluble (AI) fraction of the wild type, which indicates an elevated chitin content. The Δ*skn*7 mutant showed a much weaker and the Δ*tcs*C mutant no such increase (Fig. 11). The elevated glucosamine level was accompanied by a drop of the glucose content in the alkali-insoluble fraction of the wild type (Fig. 11) indicating a reduced β-1,3-glucan content of fludioxonil-treated wild type hyphae.

The increased expression of genes that are implicated in the synthesis of α-1,3-glucan was not reflected by the biochemical data, since the glucose content of the AI fractions of the wild type and the Δ*skn*7 mutant were rather decreasing (Fig. 11). The galactose and galactosamine moieties of GAG are expected in the AI fractions. The fludioxonil-induced increase in the expression of GAG-related genes in the wild type and the Δ*skn*7 mutant correlates only in the latter with an elevated AI galactose and AI galactosamine content, whereas a strong decrease of the AI galactosamine content was found for the wild type (Fig. 11).

**Fig. 12:**
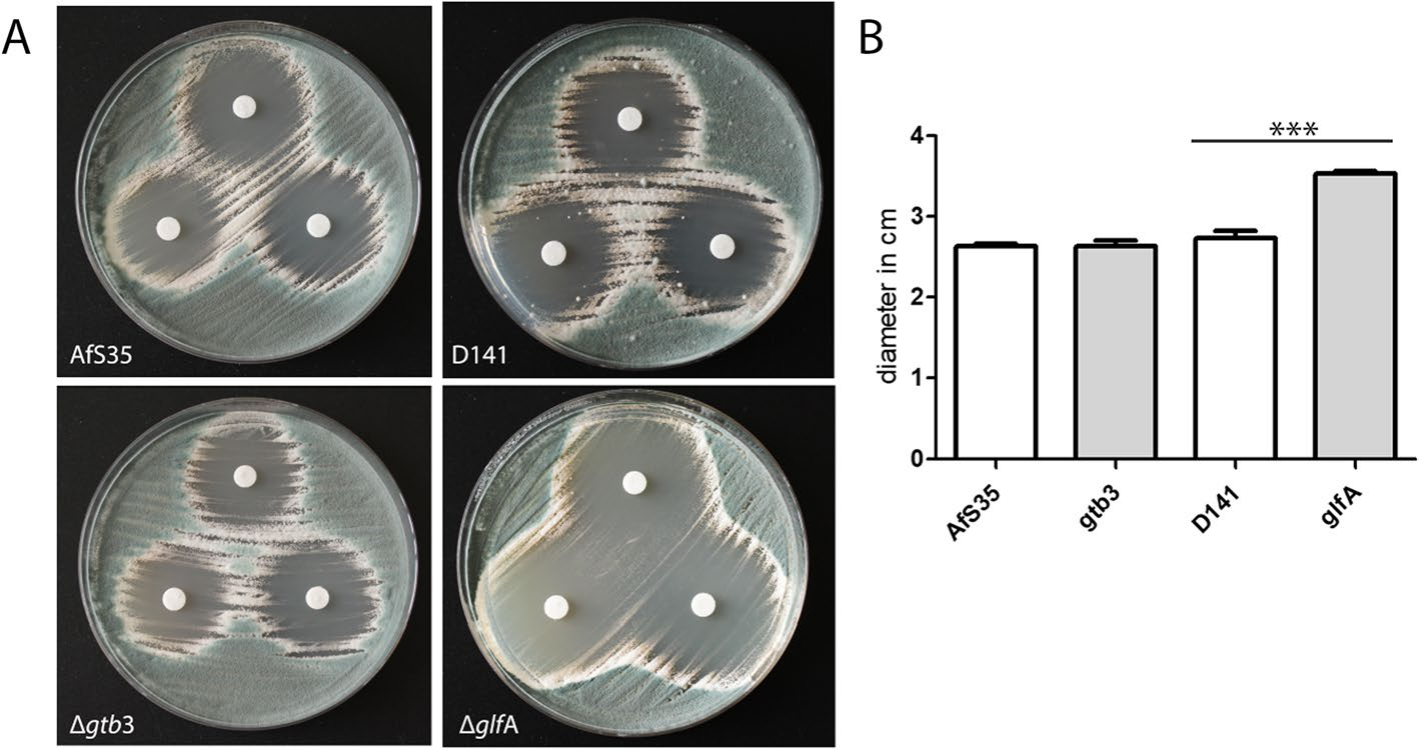
Galactomannan contributes to the resistance of *A. fumigatus* to fludioxonil. Two cell wall mutants, Δ*gtb*3 and Δ*glf*A that encode essential proteins for the synthesis of GAG and galactomannan, respectively, were analysed for their resistance to fludioxonil and compared with their parental strains AfS35 and D141, respectively. **A** inhibition zones obtained with three paper disks per plate containing 1.5 µg fludioxonil and incubated for 48 h at 37 °C. **B** the diameters of the inhibition zones were measured for three zones per strain; standard deviations are indicated

To further analyse the relevance of GAG for the impact of fludioxonil, we tested a mutant lacking the GAG synthase gene *gtb*3. This mutant was generated in AfS35; but a similar mutant was previously described for another strain [34]. As expected, the ∆*gtb*3 mutant lacked surface-accessible GAG and was unable to attach to a glass or plastic surfaces. In a paper disk assay, the fludioxonil-induced inhibition zones of the wild type and the Δ*gtb*3 mutant were comparable (Fig. 12A and B). We also tested a ∆*glf*A mutant, which lacks an essential gene for the biosynthesis of galactomannan [35]. The inhibition zones of this mutant were clearly larger than those of the wild type (Fig. 12A and B) indicating that a loss of galactomannan renders *A. fumigatus* more prone to fludioxonil.

**Fig. 13:**
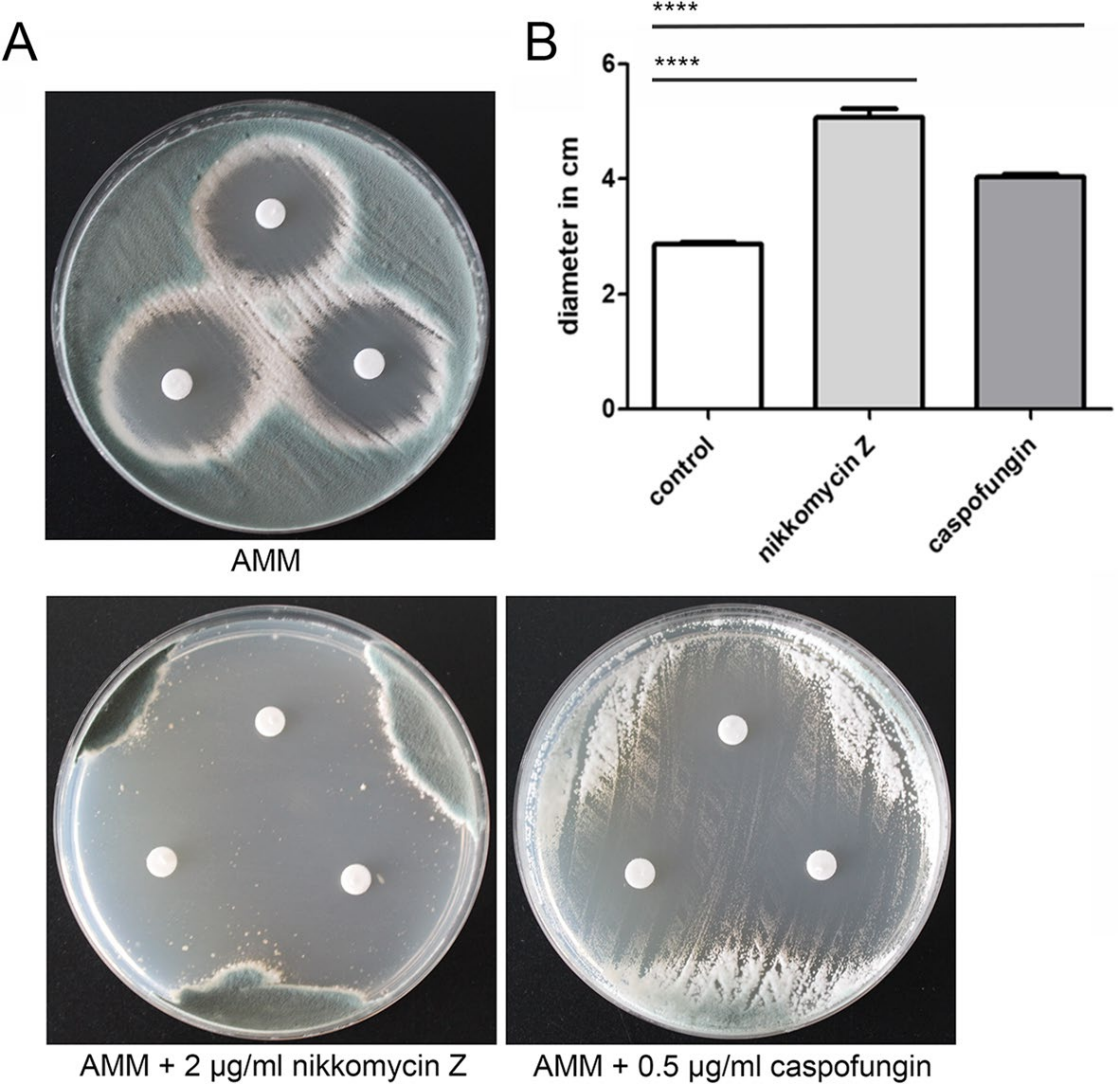
The presence of nikkomycin Z and caspofungin enhance the antifungal activity of fludioxonil. A disk diffusion assay was performed with strain AfS35 on AMM plates either without further supplement or containing sublethal concentrations of nikkomycin Z (2 µg/ml) or caspofungin (0.5 µg/ml). The paper disks were loaded with 1.5 µg fludioxonil. The plates were incubated at 37 °C and images were taken after 72 h. The diameters of the inhibition zones were measured for three zones per strain; standard deviations are indicated. **** *p* < 0.0001

Since many results of this study indicate that the antifungal activity of fludioxonil is tightly linked to the cell wall architecture, we analyzed whether the cell wall targeting drugs caspofungin and nikkomycin Z interfere with the antifungal activity of fludioxonil. To this end, we measured the inhibition zones that were formed around fludioxonil-containing paper disks on plates supplemented with sublethal concentrations of caspofungin and nikkomycin Z. The presence of both compounds resulted in larger inhibition zones. The impact of nikkomycin Z, which inhibits the synthesis of chitin, was stronger than that of caspofingin, which blocks the synthesis of β-1,3-glucan (Fig. 13).

## Discussion

Fludioxonil is a derivative of the natural antifungal pyrrolnitrin and the paradigm of a group of antifungals that activates the HOG signalling pathway [36]. This pathway consists of a MAP kinase cascade and a fungal-specific multistep phosphorelay that is targeted by these antifungals. Fludioxonil is in vitro effective against *A. fumigatus* and several other major fungal pathogens [6]. Fludioxonil-derived compounds are therefore interesting candidates to replenish the armoury of therapeutic antifungals. Ross et al. [37] recently reported that the microenvironment in the lung of patients suffering from cystic fibrosis (CF) selects for *A. fumigatus* strains harbouring a hyperactive Pbs2, the mitogen-activated protein kinase kinase of the HOG pathway. Strains harbouring this mutation had an enhanced resistance to hyperosmotic stress under oxygen limitation, which provides an advantage in the milieu of the CF lung. Remarkably, these mutations rendered the fungus also more susceptible for fludioxonil.

In the current study, we have performed an RNA-seq study to analyse the transcriptional response of *A. fumigatus* to fludioxonil and to identify genes that are implicated in the antifungal activity of this compound or that are involved in potential rescue mechanisms. Apart from the wild type, we included two mutant strains: the Δ*skn*7 mutant, showing a partial resistance to fludioxonil and pyrrolnitrin, and the completely resistant Δ*tcs*C mutant. We observed a broad transcriptional response in the wild type, a limited response in the Δ*skn*7 and a very weak response in the Δ*tcs*C mutant. Thus, the transcriptional changes matched the individual levels of sensitivity. A PCA plot (Fig. 1) shows that all three strains had a very similar transcriptional profile in the absence of fludioxonil, but the presence of this drug changed this dramatically. After 1 h and 3 h, the strains were clearly separated according to their respective resistance levels. The PCA plot also shows the very limited response of the Δ*tcs*C mutant to this antifungal.

Three *A. fumigatus* genes were previously identified to be strongly up-regulated in response to fludioxonil [3]. These genes, *cat*A, *dpr*A and *dpr*B, encode proteins that are implicated in the response to oxidative stress. They showed a similar regulation in our study, but the absolute expression levels of *drp*A and *cat*A in the wild type were only weak. In another study, Hagiwara et al. [15] described 40 *A. nidulans* genes that were strongly reactive to fludioxonil. We identified *A. fumigatus* orthologs for 37 of them and 16 of them showed a similar response in *A. fumigatus*. In both data sets, carbohydrate-related and transporter genes are up- and genes involved in RNA processing and ribosome formation are down-regulated. The fact that the orthologs of the other *A. nidulans* DEGs showed no differential expression may reflect that samples were taken at different time points (15 min versus 1 h and 3 h after fludioxonil treatment) and/or that different methods were used to analyse the transcriptional response (RNA-seq versus DNA microarray). Another important aspect that may also contribute to these differences is that the homologous response regulators Skn7 (in *A. fumigatus*) and SrrA (in *A. nidulans*) are of different relevance for the impact of fludioxonil, since Skn7 is, compared to SrrA, much more important in this context [8].

The transcriptional profiles of non-treated control hyphae of the three strains were very similar, but some differences are remarkable. A prominent example is the reduced expression of the mycelial catalase 1 (Afu3g02270) in the Δ*skn*7 mutant, a finding that fits well to the particularly high sensitivity of this mutant to hydrogen peroxide [8]. The ∆*tcs*C-specific down-regulation of the adjacent genes Afu2g17820, Afu2g17830 and Afu2g17840 is also notable. Afu2g17230 contains a domain with predicted O-methyltransferase activity and Afu2g17840 is a putative major facilitator superfamily (MFS) transporter. All three genes are part of a gene cluster that has been linked to the biosynthesis of the antibacterial alkaloid fumigaclavine [26].

TcsB is the ortholog of Sln1p and therefore potentially connected to the HOG pathway of *A. fumigatus*. The slightly stronger expression of *tcs*B (Afu2g00660) in the ∆*tcs*C mutant could reflect a compensatory response to the loss of TcsC. Whether there is indeed a functional or regulatory linkage between both proteins/genes is the subject of on-going research.

**Fig. 14:**
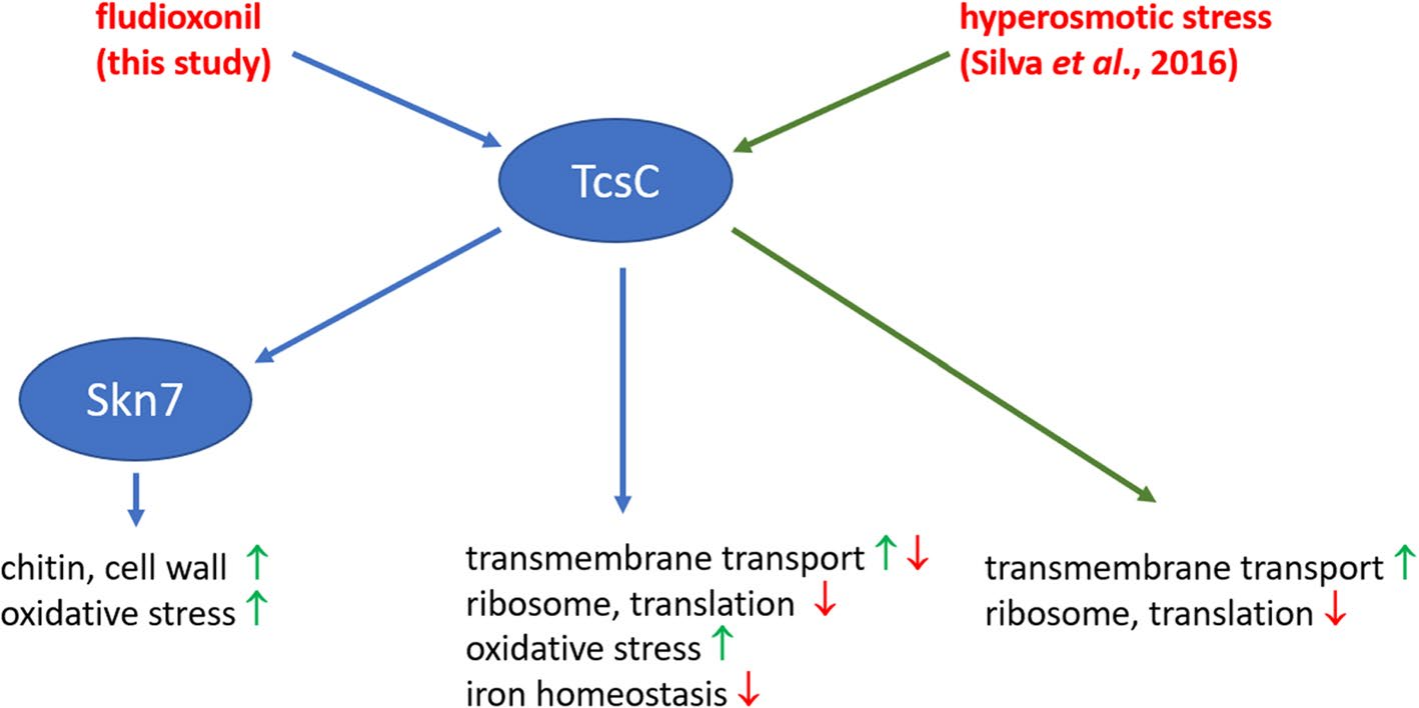
This model summarizes some key aspects of the transcriptional response of *A. fumigatus* to fludioxonil and integrates data from Pereira Silva et al. [16] on the response to hyperosmotic stress. The up- and down-regulation of the listed GO terms is indicated by green and red arrows, respectively

Fludioxonil and related agents activate group III HHKs and thereby mimic a hyperosmotic stress situation [36]. This type of response was previously analysed in *A. fumigatus* by Pereira Silva et al. [16]. A comparison of both data sets revealed a common core comprising approximately 9% of the up- and 21% of the down-regulated genes. An enhanced transcription was found for genes encoding proteins involved in carbohydrate metabolism, whereas the down-regulated genes were particular enriched for those involved in ribosome biogenesis and translation, which constitutes a typical stress response pattern. Fludioxonil triggers the up-regulation of many genes that are implicated in the biosynthesis and reorganization of cell wall carbohydrates. The set of fludioxonil-specific down-regulated genes comprises additional genes implicated in ribosome biogenesis and translation. In conclusion, these data indicate that fludioxonil triggers a pronounced stress response that includes many cell wall-related genes that are not differentially expressed upon exposure to hyperosmotic stress (Fig. 14).

Only few genes showed a similar regulation in all three strains in response to fludioxonil. We found 36 commonly regulated DEGs after 1 h and 34 after 3 h; only 4 of them showed a consistent up- and one a consistent down-regulation at both time times. These genes are most likely not important for the antifungal impact of fludioxonil. However, this finding indicates that fludioxonil triggers also certain responses that are independent of TcsC and Skn7. It needs to be mentioned that the levels of differential expression of these commonly regulated genes varied substantially, being strongest in the wild type and weakest in the Δ*tcs*C mutant; this indicates that TcsC and Skn7 nevertheless contribute to the regulation of these genes. The fact that none of these genes showed a response to hyperosmotic stress [16] indicates that they are specifically regulated by fludioxonil.

The genes that are up-regulated in all strains after 1 h comprise the most strongly regulated genes in the wild type: an IgE-binding protein (Afu6g00430), a protein of unknown function (Afu6g00690) and the putative cell wall-protein PhiA (Afu3g03060). The latter shares homology with the stress-induced major cell wall protein Cwp1p of *S. cerevisiae* and its *A. nidulans* ortholog was shown to be essential for the development of phialides [38]. These three genes were not regulated in response to hyperosmotic stress [16], but showed strong transcriptional responses in other studies, e.g. in hyphae treated with Congo red [39] or caspofungin [40]. The biological significance of this striking regulation is still elusive.

Fludioxonil activates the HOG pathway, but only two of the corresponding genes were differentially expressed in response to the antifungal. The *ypd*1 gene was moderately up-regulated in the wild type and the Δ*skn*7 mutant, whereas *tcs*B showed a wild type-specific down-regulation. The latter may reinforce the impact of fludioxonil, since TcsB is, in analogy to Sln1p, supposed to keep the HOG pathway in an inactive state.

The characteristic swelling is a hallmark of the antifungal activity of fludioxonil. It is generally assumed that an increase of the internal glycerol concentration drives this process and in *A. fumigatus* this increase is largely, but not exclusively dependent on TcsC [8]. In *A. nidulans,* the gene of the G-3-P dehydrogenase (*gfd*B), which catalyses the production of glycerol from dihydroxyacetone phosphate is strongly up-regulated in response to fludioxonil [7, 15]. In *A. fumigatus*, we observed no differential expression for the orthologous gene Afu2g08250. Instead we found an up-regulation of Afu4g11540, which encodes a putative glycerol kinase gene. One way to assimilate glycerol is the phosphorylative catabolic pathway in which glycerol becomes phosphorylated by a glycerol kinase to yield G-3-P. Afu4g11540 is strongly up-regulated in response to fludioxonil and hyperosmotic stress [16]. A mutant lacking this gene was unable to grow on glycerol, but its sensitivity to fludioxonil was not affected. In conclusion, our data imply that the antifungal activity of fludioxonil is neither determined by an overexpression of certain components of the HOG pathway nor of proteins that mediate the production of glycerol. Instead, the fludioxonil-induced response seems to get along with the HOG- and the glycerol-related proteins that are already present in the target cell. A similar mechanism likely operates in cells that experience a sudden shift of the external osmolarity. This stress situation requires an expeditious response to avoid cellular damage, which is best achieved by an activation of already existing proteins. In line with this concept, Westfall et al. found that the paramount role of *S. cerevisiae* Hog1p in the response to hyperosmotic stress is not based on a transcriptional response, but rather on the activation of proteins that establish metabolic conditions for an elevated glycerol production [41]. Whether the common regulation of several metabolic genes in the responses to fludioxonil and hyperosmotic stress, e.g., isocitrate lyase and malate dehydrogenase, reflects such a metabolic re-programming remains to be analysed.

Many DEGs found in the current study are involved in metabolic processes or biosynthetic pathways. A prominent example is a cluster of four genes that are engaged in the metabolism of ethanol. These genes are largely or exclusively wild type-specific FUGs and Afu7g01000 and Afu7g01010 belong to the most strongly up-regulated genes. Adh1 (Afu7g01010) is the ortholog of *C. albicans* Adh1, which was shown to catalyse the oxidation of methylglyoxal to pyruvate [20]. This is of particular interest, since Brandhorst et al. showed that fludioxonil interacts with the triose phosphate isomerase of *B. dermatitidis* to generate methylglyoxal, which in turn activates the group III HHK Drk1 [21]. An up-regulation of Adh1 in *A.* *fumigatus* could antagonize this by an increased enzymatic conversion of methylglyoxal. However, a deletion of the *adh*1 gene in *A. fumigatus* had no obvious impact on the sensitivity to fludioxonil. The mutant was unable to grow on ethanol indicating that Afu7g01010 is an ethanol dehydrogenase that, in collaboration with the putative acetaldehyde dehydrogenase Afu7g01010, feeds ethanol into the fungal metabolism. These genes are also strongly expressed in other stress situations, e.g., in the presence of caspofungin or hydrogen peroxide [40, 42], but there is currently no evidence that this response provides a protection to fludioxonil-treated wild type hyphae. If methylglyoxal is a key player that is induced by fludioxonil to activate TcsC, the wild type should be more sensitive to methylglyoxal than the Δ*tcs*C mutant, but drop dilution experiments provided no evidence to support this.

A remarkable, fludioxonil-induced and wild type-specific transcriptional response is the reduced expression of enzymes of the citrate-malate-shuttle and the concomitant up-regulation of genes encoding components of the glyoxylate cycle. In combination, this constitutes a fludioxonil-induced metabolic re-programming of the basic metabolism. The former suggests a reduced transport of acetyl-CoA from the mitochondria to the cytosol and the latter an increased production of citrate from acetyl-CoA in the glyoxysomes. This pattern resembles a starvation response and suggests a reduced fatty acid biosynthesis from acetyl-CoA in fludioxonil-treated cells. Alternatively, this differential expression may reflect an enhanced need for carbohydrates due to the fludioxonil-induced cell wall remodelling.

Previous studies already demonstrated fludioxonil-induced changes in the cell wall of *A. fumigatus* and our RNA-seq data demonstrate that fludioxonil triggers transcriptional responses that affect the three major cell wall carbohydrates of *A. fumigatus*: β-1,3-glucan, α-1,3-glucan and chitin. The data also show that these changes were most prominent in the wild type.

Our biochemical analysis revealed a drop in the glucose content of the AI fraction of fludioxonil-treated wild type hyphae. This indicates a reduced β-1,3-glucan content, which fits well to previous immunofluorescence data demonstrating a weaker β-glucan-specific staining of fludioxonil-treated hyphae [29]. In line, we observed a weak and wild type-specific down-regulation of the β-1,3-glucanosyltransferase gene *gel*2 after 3 h. This is remarkable since a *gel*2 deletion entails cell wall rearrangements that are reminiscent of fludioxonil-treated cells, namely a decreased β-1,3-glucan and galactomannan content and elevated levels of chitin [43]. The RNA-seq data furthermore indicate a strong up-regulation of *gel*1, *gel*5 and *gel*7 that belong to a family of seven β-1,3-glucanosyltransferase genes (*gel*1-*gel*7). Gel2 has a proven role in β-glucan biosynthesis, whereas a disruption of *gel*1 led to no discernible phenotype [43]. Gastebois et al*.* reported that *gel*5 is not expressed in *A. fumigatus* hyphae [44] and our data indeed show a very weak expression level at 0 h. However, transcription was weakly induced after 1 h and strongly up-regulated after 3 h in the presence of fludioxonil. Gel7 has been implicated in a compensatory response to cell wall stress [45]; accordingly, the late responses of *gel*5 and *gel*7 may indicate that both proteins are produced to counteract a fludioxonil-induced loss of β-1,3-glucan from the cell wall.

A strongly enhanced staining with the chitin-specific dye Calcofluor white is a hallmark of the fludioxonil-induced cell wall reorganizations [6] that is not detectable in the Δ*skn*7 mutant [8]. A dramatic wild type-specific increase of chitin is also demonstrated by our biochemical data. The chitin polymer consists of N-acetyl glucosamine moieties, and we found a strong increase of the glucosamine content of the AI fraction. In line, we also observed a strong and sustained up-regulation of the glucosamine-fructose-6-phosphate aminotransferase (Afu6g06340). This enzyme mediates the first and rate-limiting step in the biosynthesis of chitin and represents the key factor to modulate the level of chitin synthesis [46]. Another observation that fits to an elevated chitin content is the sustained and wild type-specific up-regulation of Afu7g02180. This gene encodes a putative UDP-N-acetylglucosamine pyrophosphorylase, the final enzyme in the synthesis of UDP-GlcNAc, the building block of the chitin polymer [47]. Hence, the fludioxonil-induced and wild type-specific increase of the chitin content is now corroborated by microscopic, biochemical and transcriptional data. The elevated amounts of chitin entail a stronger production of three chitosanases. These enzymes cleave chitosan, a de-acetylated derivative of chitin. Increasing amounts of chitosan likely occur in the cell wall of fludioxonil-treated hyphae and the chitosanases may be engaged in the further processing of this polymer.

The RNA-seq data indicate that genes involved in the synthesis of GAG are also up-regulated in response to fludioxonil; but at least for the wild type, our biochemical data provide no supporting evidence for this. To pursue this further, we generated a mutant lacking Δ*gtb*3, an essential gene of the GAG biosynthetic pathway. The sensitivity of this mutant to fludioxonil was similar to the wild type, indicating that GAG does not contribute to the stability of the cell wall. In contrast, we observed a strongly increased sensitivity for a Δ*glf*A mutant that lacks an essential enzyme for the biosynthesis of galactomannan. A characteristic feature of fludioxonil-treated *A. fumigatus* hyphae is the marked shedding of galactomannan [6]. The phenotype of the Δ*glf*A mutant indicates that a loss of galactomannan renders the cell wall more prone to the deleterious activity of fludioxonil. This suggests that hyphae that lost galactomannan due to the shedding may have a reduced ability to resist the fludioxonil-induced cell wall damage. Since the shedding occurs only in the wild type, this may also explain why higher levels of GAG-related enzymes can be corroborated by biochemical evidence for the Δ*skn*7 mutant, but not for the wild type. We also tested whether the cell wall targeting drugs caspofungin and nikkomycin Z interfere with the activity of fludioxonil. Sublethal doses of nikkomycin Z and caspofungin enhanced the antifungal activity of fludioxonil. The impact of nikkomycin Z was particularly strong indicating that the high amounts of chitin typically found in fludioxonil-treated wild type hyphae most likely represent a protective response that helps to stabilize the cell wall.

The differential gene expression found in the three strains indicates that fludioxonil interacts with TcsC to trigger a many-faceted outcome that mainly affects the wild type and the Δ*skn*7 mutant (this is schematically summarized in Fig. 14). Evidence mounts for a transient crisis that results in a down-regulation of genes involved in translation, ribosome biogenesis and iron homeostasis. This pattern is prominent after 1 h, but disappears later on. A distinct response pattern is evident for the iron-related genes, they show an initial down-regulation that is followed by a strong up-regulation after 3 h. The transient down-regulation of iron-related genes likely aggravates oxidative stress [42] and triggers an increased transcription of the corresponding genes. Evidence for oxidative stress exists in the wild type and the Δ*skn*7 mutant. This is particular remarkable for the latter strain, since Skn7 is functionally linked to the oxidative stress response. The largely Δ*skn*7-specific up-regulation of the two major catalase genes is particular striking in this context. At 0 h, the Δ*skn*7 mutant produces lower amounts of *cat*1 transcripts than the wild type, but after fludioxonil treatment, both major catalases are over-expressed, which suggests that after fludioxonil-treatment, the Δ*skn*7 mutant may be even better protected against hydrogen peroxide than the wild type.

## Conclusions

All transcriptional evidence suggests that fludioxonil applies a stress to the wild type and the Δ*skn*7 mutant. The relative resistance of the Δ*skn*7 mutant indicates that this strain lacks an essential and lethal part of the fludioxonil-induced response. A key signature in this context is the strong, wild type-specific up-regulation of many cell wall-related genes, in particular those involved in the biosynthesis of chitin. Ties between Skn7p and the cell wall have already detected in *S. cerevisiae* [48] and it is reasonable to assume a similar situation for *A. fumigatus*. Further research is clearly required to define the links between Skn7 and the cell wall of *A. fumigatus* at the molecular level. The resulting data should enable us to verify whether the fludioxonil-induced cell wall rearrangements are essential for the lethal impact of this agent.

## Material and methods

### Strains used in this study

The *A. fumigatus* strain AfS35 is a nonhomologous end-joining-deficient variant of the clinical isolate D141 [49]. Apart from this, AfS35 has no discernible phenotype and is therefore used as a wild type strain in this study. The corresponding deletion mutants lacking either *tcs*C (Δ*tcs*C) or *skn*7 (Δ*skn*7) have been described in previous studies [5, 8].

### RNA-seq analysis

The two mutant strains and their parental strain AfS35 were grown in AMM (1x10^6^ conidia/ml inoculum) for 18 h. For fludioxonil treatment, cultures were supplemented with 2 µg/ml fludioxonil for 1 or 3 h, while the control cultures received only the solvent control. We used a fludioxonil stock solution of 2 mg/ml fludioxonil in DMSO. For harvesting, the hyphal biomass was separated from the culture supernatant using Miracloth (Merck Millipore, Darmstadt, Germany) and dried between paper towels. It was flash-frozen in liquid nitrogen and ground with mortar and pestle under liquid nitrogen to release the cellular content. RNA was isolated from the resulting powder using the Qiagen Plant RNeasy Mini Kit (Hilden, Germany) according to the manufacturer’s instructions. RNA concentrations were quantified using a NanoDrop ND-1000 UV VIS spectrophotometer (Thermo Fisher Scientific, Waltham, USA) at 260 nm and samples were stored at -80 °C until use. The total RNA was used for Illumina sequencing (Illumina NextSeq 500 V2) and processed by LGC Genomics (Berlin, Germany) using 75 bp single reads.

### Data pre-processing

Raw data were pre-processed using the RNA-seq pre-processing pipeline “GEO2RNAseq” package [50] in R version 3.2.0. Briefly, low-quality reads and adapters were removed using Trimmomatic version 0.36 [51]. rRNA reads were removed using SortMeRNA version 2.1 [52]. High-quality reads were mapped against the *A. fumigatus* Af293 reference genome (version s03-m05-r11) obtained from the Aspergillus Genome Database using HISAT2 version 2.1.0 [53]. Read counts were estimated using Rsubread's feature Counts version 1.20.6 [54]). The “Median Ratio Normalization” (MRN) method was employed to normalize read counts across all samples. Principal component analysis (PCA) was performed using “prcomp” functions from the “stats” package in R (R core team, 2015).

DEGs were calculated for 15 comparisons focussing on differences in gene expression between time points and between strains. The significant DEGs were tested by four different packages in R including DESeq version 1.22.1, DESeq2 version 1.10.1 [55], edgeR version 3.12.1 [56], and limma version 3.26.9 [57]. An absolute log_2_FC of 1.5 and an FDR-adjusted *p*-value of 0.01 were applied as cut-off. The final DEG list is determined by deriving the intersection of significant DEGs reported by all four aforementioned tools.

The RNA-seq data of this study are available at the database repository Gene Expression Omnibus (GEO) with the accession number GSE224696. The data set was further analyzed with the Gene Ontology Enrichment tool using GO Slim at https://fungiDB.org. Enriched GO terms of the ontology “Biological process” were searched using a p-value threshold of 0.05. Gene sets were compared using Venny 2.1.0 (https://bioinfogp.cnb.csic.es/tools/venny/).

### Analysis of differential gene expression by qPCR

RNA isolation from fungal samples was performed as described above. cDNA was synthesized using the High-Capacity cDNA Reverse Transcription Kit (ThermoFisher, Waltham, MA, USA) and qPCRs were performed with 5X EvaGreen®Mastermix (Bio&Sell, Feucht, Germany) using a QuantStudio5 qPCR cycler (ThermoFisher). After initial denaturation for 15 min at 95 °C, 40 PCR cycles were performed (denaturation for 15 s at 95 °C, annealing for 20 s at 60 °C and elongation for 20 s at 72 °C). Expression levels of the corresponding genes in the presence of fludioxonil were normalized to the expression of the *tub*A gene and the expression levels of untreated controls according to the ΔΔCt method. Oligonucleotides used for qPCRs are listed in Additional Table 4.

### Generation and characterization of mutant strains

The oligonucleotides used to generate the deletion cassettes for Afu7g01010 (*adh*1), Afu4g11540 (glycerol kinase) and Afu3g07860 (GAG transmembrane glycosyltransferase) and to screen the resultant mutant strains are summarized in Additional Table 4. Approximately 1000 bp regions up- and downstream of the respective target gene were amplified with primers containing suitable SfiI sites to ligate the PCR products to a 4.8 kb hygromycin B resistance cassette. This cassette was excised from plasmid pSK528 by SfiI digestion. (Plasmid pSK528 was kindly provided by Sven Krappmann, Erlangen).

Protoplast transformation was used to introduce the resulting deletion cassette into strain AfS35. The presence of the resistance cassette and the deletion of the respective target gene in the clones that grew on hygromycin plates were verified by PCR using the oligonucleotides listed in Additional Table 4. Phenotypic testing using drop dilution or paper disk assays and the quantification of glycerol concentrations were performed as described previously [8]. For disk diffusion assays, AMM plates containing either 2 µg/ml nikkomycin Z, 0.5 µg/ml caspofungin or no additional compound were homogeneously inoculated with a swab from a suspension of AfS35 conidia (2 × 10^7^/ml). Paper disks were placed on the plates and loaded with 1.5 µg fludioxonil. Agar plates were incubated for 72 h at 37 °C. Inhibition zones were measured using a Scan 4000 device (Interscience, Saint Nom la Bretèche, France).

### Cell wall analysis

Fungal mycelia were obtained by inoculating 2 × 10^8^ conidia of each strain into 100 ml liquid culture medium (AMM) either with or without fludioxonil (2 µg/ml). These cultures were grown for 24 h at 30 °C and 140 rpm. Mycelia thus formed were collected by centrifugation (5000 rpm, 10 min) and washed thrice with MilliQ water. Mycelia were disrupted using glass beads (0.75–1 mm) in a FastPrep (MP Biomedicals), the cell wall fraction was collected by centrifugation and washed thrice with MilliQ water. Following, this cell wall fraction was subjected to Tris (50 mM)-EDTA (50 mM)-sodium dodecyl-sulphate (2%)-β-mercaptoethanol (40 mM) buffer (pH 7.4) treatment in a boiling-water bath, twice, discarding the supernatant obtained after centrifugation after each boiling step; this is to separate bound or entangled proteins in the cell wall, to obtain only the polysaccharide fraction. The cell wall polysaccharide mixture thus obtained was subjected to alkali-fractionation upon incubating it in 1 M NaOH containing 0.5 M NaBH_4_ in a water-bath maintained at 70 °C, twice, each time separating the pellet [alkali-insoluble (AI) fraction] from the supernatant [alkali-soluble (AS) fraction]. The AI-fraction was washed till neutrality, whereas the AS-fraction was dialyzed against water until neutrality; both fractions were then freeze dried. Further, AI and AS fractions were acid hydrolysed, derivatized and subjected to gas chromatography analysis to determine their monosaccharide composition, following the protocol described earlier [58]. The cell wall analysis was performed on three biological replicates of the mycelia, for each fungal strain.

### Supplementary Information

**Additional file 1: Additional Table 1. **Regulation of the 40 most strongly differentially expressed A. nidulans genes in response to fludioxonil and their A. fumigatus orthologs.** Additional Table 2. **Genes that are differentially expressed in the absence of fludioxonil. **Additional Table 3. **Genes showing a wild type-specific differential expression after 1 and 3 h. **Additional Table 4. **Oligonucleotides used in this study.  

## Data Availability

The RNA-seq data set is available at the Gene Expression Omnibus (GEO) database repository with the accession number GSE224696.
