# Supplementary material for: Distinct transcriptional responses to fludioxonil in Aspergillus fumigatus and its ΔtcsC and Δskn7 mutants reveal a crucial role for Skn7 in the cell wall reorganizations triggered by this antifungal
Source: BMC Genomics. 2023 Nov 14;24:684. doi: 10.1186/s12864-023-09777-5 (PMC10647056; doi:10.1186/s12864-023-09777-5)
Supplement: Supplementary file 1 — Additional file 1: Additional Table 1. Regulation of the 40 most strongly differentially expressed A. nidulans genes in response to fludioxonil and their A. fumigatus orthologs. Additional Table 2. Genes that are differentially expressed in the absence of fludioxonil. Additional Table 3. Genes showing a wild type-specific differential expression after 1 and 3 h. Additional Table 4. Oligonucleotides used in this study. [file 12864_2023_9777_MOESM1_ESM.docx]

**Additional Table 1: Regulation of the 40 most strongly differentially expressed *A. nidulans* genes in response to fludioxonil and their *A. fumigatus* orthologs.**

**A: Fludioxonil-responsive genes in in *A. nidulans*: The 20 most strongly up-regulated genes according to [15]**

|  |  | **log_2_FC** |  | **log_2_FC** | |
| --- | --- | --- | --- | --- | --- |
| **Identifier** | **Description** | ***A. nidulans*** | **Identifier** | **wt 1 h vs 0 h** | **wt 3 h vs 0 h** |
| AN3358 | endo-1,4-beta-mannosidase | 7.50 | Afu8g07030 | -- | -- |
| AN7390 | MFS multidrug transporter | 6.72 | Afu6g13780 | -- | -- |
| AN4277 | MFS monosaccharide transporter | 6.66 | **Afu7g00950** | 4.17 | 3.93 |
| AN8339 | hypothetical protein | 6.45 | **Afu7g06770** | 2.58 | 2.44 |
| AN1301 | extracellular dioxygenase | 6.27 | Afu6g03070 | -- | -- |
| AN2792 | hypothetical protein | 6.13 | Afu8g02480 | -- | -- |
| AN6792 | glycerol-3-phosphate dehydrogenase GfdB | 6.10 | Afu2g08250 | -- | -- |
| AN3357 | low-affinity glucose transporter MstE | 6.02 | **Afu7g00950** | 4.17 | 3.93 |
| AN8930 | C6 transcription factor | 5.77 | Afu3g10160 | -- | -- |
| AN6835 | P450 family fatty acid hydroxylase | 5.72 | Afu3g09220 | -- | -- |
| AN8195 | protein of unknown function | 5.50 | n.o. |  |  |
| AN9173 | MFS glucose transporter | 5.48 | Afu3g14170 | -- | -- |
| AN9168 | MFS sugar transporter Stl1 | 5.46 | **Afu8g05710** | 2.52 | 2.98 |
| AN7959 | hypothetical protein | 5.41 | Afu5g12830 | -- | -- |
| AN5624 | hypothetical protein | 5.41 | Afu4g11110 | -- | -- |
| AN8628 | putative NADP-dependent alcohol dehydrogenase | 5.39 | **Afu6g00510** | 2.26 | 1.75 |
| AN10303 | mitochondrial carrier protein | 5.33 | Afu2g13870 | -- | -- |
| AN11016 | malic acid transport protein | 5.33 | **Afu4g09410** | -- | 1.55 |
| AN11314 | hypothetical protein | 5.28 | n.o. |  |  |
| AN11495 | hypothetical protein | 5.22 | n.o. |  |  |

**B: Fludioxonil-responsive genes in in *A. nidulans*: The 20 strongly down-regulated genes according to [15]**

|  |  | **log_2_FC** |  | **log_2_FC** | |
| --- | --- | --- | --- | --- | --- |
| **Identifier** | **Description** | ***A. nidulans*** | **Identifier** | **wt 1 h vs 0 h** | **wt 3 h vs 0 h** |
| AN1666 | nucleolar GTPase | **-**5.28 | **Afu4g08930** | **-**2.61 | -- |
| AN7877 | hypothetical protein | -4.58 | Afu2g17810 | 1.93 | 3.20 |
| AN6006 | importin Nmd5 | -4.43 | Afu2g10010 | -- | -- |
| AN4581 | essential nuclear protein Sgd1 | -4.42 | **Afu2g01980** | -2.35 | -- |
| AN9076 | hypothetical protein | -4.32 | Afu7g02390 | -- | -- |
| AN2084 | hypothetical protein | -4.19 | Afu2g04900 | -- | -- |
| AN1711 | nonsense-mediated mRNA decay protein 3 | -4.06 | Afu2g16750 | (-1.49) | -- |
| AN6221 | GATA transcription factor AreB alpha | -4.03 | Afu2g13380 | -- | -- |
| AN8183 | small nucleolar ribonucleoprotein complex subunit | -4.01 | **Afu5g03090** | -1.91 | -- |
| AN7609 | mitochondrial DNA-directed RNA polymerase | -3.97 | Afu2g15610 | (-1.45) | -- |
| AN3048 | hypothetical protein | -3.94 | Afu2g07930 | -- | -- |
| AN0054 | adenylate-forming enzyme | -3.88 | Afu5g12510 | -- | -- |
| AN4330 | ribosome biogenesis (Nop4) | -3.76 | **Afu4g06250** | -2.48 | -- |
| AN7554 | oxidoreductase | -3.71 | **Afu2g14810** | -2.06 | -- |
| AN7540 | eukaryotic translation initiation factor 3 subunit Moe1 | -3.68 | **Afu2g14670** | -1.57 | -- |
| AN4233 | ATP-dependent RNA helicase | -3.67 | **Afu1g06220** | -3.10 | -- |
| AN8491 | 60S ribosome biogenesis protein | -3.66 | **Afu2g17060** | -2.93 | -- |
| AN1367 | ribosome biogenesis protein | -3.64 | **Afu1g09200** | -2.35 | -- |
| AN6244 | component of the exosome 3 → 5 exonuclease complex | -3.60 | Afu2g13130 | -- | -- |
| AN9381 | hypothetical protein | -3.59 | Afu2g07930 | -- | -- |

n.o. = no orthologous gene found

**Additional Table 1:** The 40 most strongly differentially expressed *A. nidulans* genes in response to fludioxonil according to [15]. The table lists the orthologous *A. fumigatus* genes and their log_2_FC values after 1 h and 3 h of fludioxonil treatment. The up-regulated gens are listed in (A) and the down-regulated genes in (B). *A. fumigatus* genes showing a similar regulation as their *A. nidulans* orthologs are indicated in bold. The description of gene function is according to [15]. (--) indicates genes with log_2_FC values below 1.2; n.o. = no orthologs found in *A. fumigatus*.

**Additional Table 2: Genes that are differentially expressed in the absence of fludioxonil**

|  |  |  |
| --- | --- | --- |
|  |  |  |
| **A** | **Δ*skn*7 versus wt** |  |
| **Identifier** | **Description** | **log_2_FC** |
| Afu7g00440 | GABA permease, putative | 3.77 |
| **Afu7g05180** | Defensin domain protein | 3.32 |
| **Afu4g13990** | conserved hypothetical protein | 2.94 |
| Afu2g17400 | C-3 sterol dehydrogenase/C-4 decarboxylase family protein | 2.82 |
| **Afu8g06980** | glycosyl hydrolase, putative | 2.58 |
| **Afu3g03060** | cell wall protein PhiA | 2.52 |
| **Afu8g07100** | conserved hypothetical protein | 2.52 |
| Afu7g00420 | hypothetical protein | 2.42 |
| **Afu6g00690** | conserved hypothetical protein | 2.18 |
| Afu2g14661 | hydrophobin, putative | 2.04 |
| **Afu6g00680** | conserved hypothetical protein | 2.02 |
| Afu8g00510 | cytochrome P450 oxidoreductase OrdA-like, putative | 1.97 |
| Afu8g00490 | PKS-like enzyme, putative | 1.87 |
| Afu2g12680 | conserved hypothetical protein | 1.81 |
| Afu6g03080 | ABC multidrug transporter, putative | 1.81 |
| **Afu7g00990** | transcriptional activator of ethanol catabolism AlcS | 1.80 |
| **Afu4g14790** | cytochrome P450 monooxygenase, putative | 1.79 |
| **Afu4g14840** | transferase family protein | 1.75 |
| Afu3g01200 | integral membrane protein Pth11-like, putative | 1.74 |
| Afu5g09580 | conidial hydrophobin Hyp1/RodA | 1.73 |
| Afu5g12690 | dihydroxyacetone kinase (DakA), putative | 1.72 |
| Afu6g03230 | cell wall glucanase/allergen F16-like | 1.71 |
| **Afu1g01190** | conserved hypothetical protein | 1.71 |
| Afu3g12120 | fatty acid oxygenase PpoC, putative | 1.67 |
| Afu7g04030 | hypothetical protein | 1.65 |
| Afu2g13265 | conserved hypothetical protein | 1.61 |
| Afu5g15060 | terpene synthase family protein | 1.60 |
| Afu1g01180 | isoamyl alcohol oxidase, putative | 1.59 |
| **Afu8g02110** | hypothetical protein | 1.58 |
| Afu1g10380 | nonribosomal peptide synthase Pes1 | -1.51 |
| Afu6g09505 | hypothetical protein | -1.51 |
| Afu7g04930 | alkaline serine protease (PR1)/allergen F18-like | -1.52 |
| **Afu6g08400** | conserved hypothetical protein | -1.56 |
| Afu3g01670 | MFS hexose transporter, putative | -1.57 |
| Afu8g01250 | GNAT family acetyltransferase, putative | -1.63 |
| Afu1g11715 | conserved hypothetical protein | -1.64 |
| Afu3g02270 | mycelial catalase Cat1 | -1.66 |
| Afu7g06330 | hypothetical protein | -1.69 |
| Afu8g06070 | hypothetical protein | -1.76 |
| Afu1g10390 | ABC multidrug transporter, putative | -1.82 |
| Afu3g00570 | conserved hypothetical protein | -2.17 |
| AfuMt00140 |  | -2.22 |
| Afu3g03290 | conserved hypothetical protein | -3.17 |
| Afu3g01650 | WSC domain protein, putative | -3.83 |
| Afu6g12522 | stress response transcription factor SrrA/Skn7, putative | -8.57 |
|  |  |  |
|  |  |  |
| **B** | **Δ*tcs*C versus wt** |  |
| **Identifier** | **Descripton** | **log_2_FC** |
| Afu3g03010 | phosphate-repressible Na+/phosphate cotransporter Pho89, putative | 3.54 |
| **Afu7g05180** | Defensin domain protein | 3.42 |
| Afu6g00430 | IgE-binding protein | 3.19 |
| Afu6g06535 | conserved hypothetical protein | 2.65 |
| Afu6g06530 | hypothetical protein | 2.59 |
| Afu2g00580 | hypothetical protein | 2.46 |
| Afu8g05805 | cellobiose dehydrogenase, putative | 2.43 |
| Afu8g00360 | NlpC/P60-like cell-wall peptidase, putative | 2.40 |
| Afu3g12910 | O-methyltransferase GliM-like, putative | 2.31 |
| Afu3g03300 | FAD-binding oxidoreductase, putative | 2.25 |
| **Afu4g14790** | cytochrome P450 monooxygenase, putative | 2.24 |
| Afu3g01650 | WSC domain protein, putative | 2.20 |
| **Afu4g14840** | transferase family protein | 2.18 |
| **Afu8g06980** | glycosyl hydrolase, putative | 2.18 |
| Afu2g17290 | hypothetical protein | 2.16 |
| Afu4g14770 | squalene-hopene-cyclase, putative | 2.13 |
| **Afu8g07100** | conserved hypothetical protein | 2.10 |
| **Afu6g00690** | conserved hypothetical protein | 2.09 |
| Afu3g03310 | RTA1 domain protein | 1.94 |
| Afu5g01360 | cytochrome P450, putative | 1.94 |
| Afu6g00140 | hypothetical protein | 1.92 |
| Afu6g01920 | xenobiotic compound monooxygenase, DszA family | 1.86 |
| **Afu4g13990** | conserved hypothetical protein | 1.86 |
| Afu4g14810 | cytochrome P450 monooxygenase, putative | 1.83 |
| Afu4g14800 | short chain dehydrogenase, putative | 1.79 |
| Afu2g04890 | TAM domain methyltransferase, putative | 1.78 |
| Afu4g14820 | transferase family protein | 1.77 |
| Afu2g12630 | allergenic cerato-platanin Asp F13 | 1.75 |
| Afu7g00970 | GPI anchored serine-threonine rich protein | 1.74 |
| **Afu7g00990** | transcriptional activator of ethanol catabolism AlcS | 1.73 |
| Afu2g00670 | protein kinase, putative | 1.73 |
| **Afu1g01190** | conserved hypothetical protein | 1.72 |
| Afu3g01630 | RTA1 domain protein, putative | 1.71 |
| **Afu8g02110** | hypothetical protein | 1.71 |
| Afu4g14780 | cytochrome P450 monooxygenase, putative | 1.69 |
| Afu8g07225 | conserved hypothetical protein | 1.67 |
| **Afu6g00680** | conserved hypothetical protein | 1.65 |
| Afu4g14640 | low affinity iron transporter, putative | 1.64 |
| Afu3g12900 | MFS transporter, putative | 1.64 |
| Afu3g07550 | DUF1275 domain protein | 1.60 |
| Afu2g12740 | methyltransferase, putative | 1.59 |
| Afu6g00180 | hypothetical protein | 1.58 |
| Afu5g00910 | MBL2-like secreted peptide, putative | 1.58 |
| Afu4g14460 | conserved hypothetical protein | 1.58 |
| Afu6g01840 | C6 transcription factor, putative | 1.57 |
| Afu4g01270 | integral membrane protein | 1.56 |
| Afu5g13730 | NlpC/P60-like cell-wall peptidase, putative | 1.53 |
| Afu2g15860 | TAM domain methyltransferase, putative | 1.53 |
| Afu4g04320 | homeobox transcription factor, putative | 1.52 |
| Afu7g06580 | FAD/FMN-containing isoamyl alcohol oxidase MreA-like, putative | 1.51 |
| **Afu3g03060** | cell wall protein PhiA | 1.51 |
| Afu4g12940 | hypothetical protein | 1.51 |
| **Afu6g08400** | conserved hypothetical protein | -1.53 |
| Afu8g00140 | MFS transporter, putative | -1.56 |
| Afu1g13830 | threonine-rich protein | -1.79 |
| Afu7g05490 | conserved hypothetical protein | -1.81 |
| Afu3g10850 | DUF821 domain protein | -1.89 |
| Afu2g17830 | hypothetical protein | -1.94 |
| Afu2g17420 | Pfs and NB-ARC domain protein | -1.94 |
| Afu6g13820 | conserved hypothetical protein | -1.96 |
| Afu2g14420 | cutinase, putative | -1.98 |
| Afu2g17840 | MFS transporter, putative | -2.02 |
| Afu2g17820 | O-methyltransferase | -2.06 |
| Afu4g13050 | conserved hypothetical protein | -2.08 |
| Afu5g01190 | conserved hypothetical protein | -2.11 |
| Afu6g00560 | hypothetical protein | -2.31 |
| Afu2g00963 | hypothetical protein | -2.49 |
| AfuMt00030 |  | -2.84 |
| Afu2g03560 | two-component osmosensing histidine kinase (Bos1), putative | -10.17 |
|  |  |  |
|  |  |  |
| **C** | **Δ*skn*7 versus Δ*tcs*C** |  |
| **Identifier** | **Description** | **log_2_FC** |
| Afu2g03560 | two-component osmosensing histidine kinase (Bos1), putative | 10.47 |
| Afu7g00440 | GABA permease, putative | 4.85 |
| Afu7g00420 | hypothetical protein | 3.47 |
| Afu7g05490 | conserved hypothetical protein | 3.19 |
| Afu6g00560 | hypothetical protein | 2.82 |
| Afu3g10850 | DUF821 domain protein | 2.66 |
| Afu5g09580 | conidial hydrophobin Hyp1/RodA | 2.54 |
| Afu2g00963 | hypothetical protein | 2.46 |
| Afu2g17820 | O-methyltransferase | 2.43 |
| Afu6g07855 | conserved threonine rich protein | 2.39 |
| Afu2g11020 | triosephosphate isomerase | 2.38 |
| Afu2g17840 | MFS transporter, putative | 2.28 |
| Afu6g13820 | conserved hypothetical protein | 2.24 |
| Afu2g17420 | Pfs and NB-ARC domain protein | 2.22 |
| Afu2g17400 | C-3 sterol dehydrogenase/C-4 decarboxylase family protein | 2.21 |
| Afu3g10860 | hypothetical protein | 2.20 |
| Afu8g00510 | cytochrome P450 oxidoreductase OrdA-like, putative | 2.04 |
| Afu5g00720 | GNAT family acetyltransferase, putative | 2.03 |
| AfuMt00030 |  | 2.02 |
| Afu2g17830 | hypothetical protein | 2.00 |
| Afu6g00555 | conserved hypothetical protein | 1.97 |
| Afu1g13830 | threonine-rich protein | 1.96 |
| Afu5g12690 | dihydroxyacetone kinase (DakA), putative | 1.91 |
| Afu3g03640 | MFS siderochrome iron transporter MirB | 1.91 |
| Afu5g01190 | conserved hypothetical protein | 1.90 |
| Afu4g13050 | conserved hypothetical protein | 1.90 |
| Afu2g00960 | NACHT domain protein | 1.87 |
| Afu5g14570 | conserved hypothetical protein | 1.85 |
| Afu6g03260 | aspartic endopeptidase (AP1), putative | 1.83 |
| Afu2g14661 | hydrophobin, putative | 1.81 |
| Afu2g17930 | integral membrane protein | 1.81 |
| Afu6g03440 | fructosyl amino acid oxidase, putative | 1.80 |
| Afu6g11810 | RTA1 domain protein, putative | 1.76 |
| Afu1g10610 | conserved hypothetical protein | 1.72 |
| Afu6g03230 | cell wall glucanase/allergen F16-like | 1.70 |
| Afu7g06620 | integral membrane protein Pth11-like, putative | 1.69 |
| Afu3g02780 | MFS multidrug transporter, putative | 1.67 |
| Afu3g03550 | hypothetical protein | 1.65 |
| Afu2g14661 | hydrophobin, putative | 1.81 |
| Afu4g11210 | pheromone-regulated multispanning membrane protein Prm1, putative | 1.64 |
| Afu6g03080 | ABC multidrug transporter, putative | 1.64 |
| Afu7g05500 | glutathione S-transferase, putative | 1.64 |
| Afu3g01200 | integral membrane protein Pth11-like, putative | 1.63 |
| Afu3g03270 | isochorismatase family hydrolase, putative | 1.61 |
| Afu2g14420 | cutinase, putative | 1.61 |
| Afu3g13500 | hypothetical protein | 1.57 |
| Afu4g02710 | conserved hypothetical protein | 1.55 |
| Afu4g14712 | C6 transcription factor, putative | 1.55 |
| Afu3g15250 | MFS drug efflux transporter, putative | 1.55 |
| Afu6g12140 | hypothetical protein | 1.53 |
| Afu1g04455 | hypothetical protein | 1.52 |
| Afu2g01370 | GABA permease, putative | 1.52 |
| Afu5g15060 | terpene synthase family protein | 1.51 |
| Afu5g01005 | conserved hypothetical protein | 1.51 |
| AfuMt00040 |  | 1.51 |
| Afu2g00530 | DUF1275 domain protein | -1.50 |
| Afu4g01140 | MFS multidrug transporter, putative | -1.52 |
| Afu7g05015 | glyoxalase family protein | -1.53 |
| Afu1g04150 | tartrate dehydrogenase, putative | -1.53 |
| Afu6g01840 | C6 transcription factor, putative | -1.53 |
| Afu3g14030 | extracellular phytase, putative | -1.57 |
| Afu5g00870 | conserved hypothetical protein | -1.57 |
| Afu4g01285 |  | -1.61 |
| Afu2g00660 | sensor histidine kinase/response regulator TcsB/Sln1, putative | -1.63 |
| Afu8g00360 | NlpC/P60-like cell-wall peptidase, putative | -1.63 |
| Afu7g02380 | ferulic acid esterase (FaeA), putative | -1.63 |
| AfuMt00140 |  | -1.64 |
| Afu2g15860 | TAM domain methyltransferase, putative | -1.65 |
| Afu4g01040 | short chain dehydrogenase, putative | -1.70 |
| Afu5g01360 | cytochrome P450, putative | -1.71 |
| Afu4g14640 | low affinity iron transporter, putative | -1.72 |
| Afu5g13160 | MFS transporter, putative | -1.72 |
| Afu8g01710 | antigenic thaumatin domain protein, putative | -1.74 |
| Afu4g08230 | aldehyde reductase, putative | -1.74 |
| Afu3g01670 | MFS hexose transporter, putative | -1.75 |
| Afu3g03310 | RTA1 domain protein | -1.77 |
| Afu8g01660 | hypothetical protein | -1.78 |
| Afu3g03770 |  | -1.79 |
| Afu7g00970 | GPI anchored serine-threonine rich protein | -1.79 |
| Afu3g01960 | conserved hypothetical protein | -1.82 |
| Afu5g01300 | integral membrane protein | -1.84 |
| Afu2g00670 | protein kinase, putative | -1.85 |
| Afu3g12900 | MFS transporter, putative | -1.86 |
| Afu2g00450 | hexokinase, putative | -1.87 |
| Afu5g00910 | MBL2-like secreted peptide, putative | -1.88 |
| Afu6g14510 | monooxygenase, putative | -1.88 |
| Afu5g08760 | hypothetical protein | -1.89 |
| Afu6g00180 | hypothetical protein | -1.89 |
| Afu3g13940 | DUF1212 domain membrane protein Prm10, putative | -1.90 |
| Afu3g13620 | cupin domain protein | -1.90 |
| Afu5g01290 | zinc-binding oxidoreductase, putative | -1.91 |
| Afu6g01920 | xenobiotic compound monooxygenase, DszA family | -1.92 |
| Afu3g07550 | DUF1275 domain protein | -1.93 |
| Afu5g10090 | 3-demethylubiquinone-9 3-methyltransferase, putative | -1.99 |
| Afu6g00430 | IgE-binding protein | -2.02 |
| Afu4g10930 | N-acetyltransferase family protein, putative | -2.07 |
| Afu8g05805 | cellobiose dehydrogenase, putative | -2.09 |
| Afu1g10390 | ABC multidrug transporter, putative | -2.16 |
| Afu3g00570 | conserved hypothetical protein | -2.17 |
| Afu3g09690 | extracellular thaumatin domain protein, putative | -2.18 |
| Afu3g12910 | O-methyltransferase GliM-like, putative | -2.18 |
| Afu3g03290 | conserved hypothetical protein | -2.18 |
| Afu2g04890 | TAM domain methyltransferase, putative | -2.35 |
| Afu1g10380 | nonribosomal peptide synthase Pes1 | -2.42 |
| Afu2g12740 | methyltransferase, putative | -2.78 |
| Afu6g06535 | conserved hypothetical protein | -2.78 |
| Afu8g01250 | GNAT family acetyltransferase, putative | -2.82 |
| Afu3g01630 | RTA1 domain protein, putative | -3.09 |
| Afu2g00580 | hypothetical protein | -3.10 |
| Afu6g06530 | hypothetical protein | -3.14 |
| Afu3g03010 | phosphate-repressible Na+/phosphate cotransporter Pho89, putative | -4.12 |
| Afu3g01650 | WSC domain protein, putative | -6.03 |
| Afu6g12522 | stress response transcription factor SrrA/Skn7, putative | -8.93 |

**Additional Table 2** List of genes that are differentially regulated in the three strains in the absence of fludioxonil. The table derives from pairwise comparisons of wild type versus Δ*skn*7 (A), wild type versus Δ*tcs*C (B) and Δ*skn*7 versus Δ*tcs*C (C).

**Additional Table 3:** **Genes showing a wild type-specific differential expression after 1 and 3 h**

|  | **Wild type-specific FUGs at both time points** | **log_2_FC** | |
| --- | --- | --- | --- |
| **Identifier** | **Designation** | **1 h vs 0 h** | **3 h vs 0 h** |
| Afu2g10130 | putative adhesin | 4.63 | 4.16 |
| Afu8g00360 | putative NlpC/P60-like cell-wall peptidase | 4.14 | 2.75 |
| Afu8g06990 | ankyrin repeat protein | 3.37 | 2.91 |
| Afu8g00420 | putative C6 finger transcription factor | 3.31 | 4.09 |
| Afu6g04690 | conserved hypothetical protein | 3.40 | 4.90 |
| Afu7g00150 | putative FAD-dependent monooxygenase | 3.27 | 1.56 |
| Afu1g01920 | putative plasma membrane protein Pth11-like | 2.67 | 3.45 |
| Afu1g17320 | putative endo-arabinanase | 2.63 | 4.24 |
| Afu5g11020 | ammonium transporter MepA | 2.74 | 1.90 |
| Afu5g01920 | putative GPI anchored protein | 2.96 | 3.68 |
| Afu8g02510 | glycosyl hydrolase family 43 protein | 2.60 | 2.35 |
| Afu8g06710 | hypothetical protein | 2.63 | 2.17 |
| Afu6g14030 | putative acyltransferase | 2.60 | 2.33 |
| Afu2g11030 | hypothetical protein | 2.55 | 2.87 |
| Afu6g07260 | putative purine-cytosine permease | 2.19 | 2.71 |
| Afu3g14200 | conserved hypothetical protein | 2.49 | 3.54 |
| Afu4g14410 | hypothetical protein | 2.53 | 2.91 |
| Afu8g05710 | putative MFS sugar transporter Stl1 | 2.53 | 2.98 |
| Afu3g14910 | putative FacC-like extracellular signaling protein | 2.51 | 1.55 |
| Afu4g03830 | conserved hypothetical protein | 2.48 | 2.28 |
| Afu4g13080 | putative MFS monosaccharide transporter | 2.55 | 3.62 |
| Afu4g01050 | putative ABC multidrug transporter | 2.46 | 1.69 |
| Afu2g17440 | putative 2-amino-3-carboxymuconate-6-semialdehyde decarboxylase | 2.16 | 2.83 |
| Afu8g00410 | putative methionine aminopeptidase type II | 2.43 | 3.10 |
| Afu8g02540 | hypothetical protein | 2.41 | 2.19 |
| Afu4g01220 | conserved hypothetical protein | 2.37 | 2.70 |
| Afu4g09700 | conserved hypothetical protein | 2.53 | 1.96 |
| Afu7g04030 | hypothetical protein | 2.35 | 3.12 |
| Afu7g06370 | putative C6 transcription factor | 2.58 | 1.86 |
| Afu7g04030 | hypothetical protein | 2.35 | 3.12 |
| Afu3g03300 | putative FAD-binding oxidoreductase | 2.29 | 3.90 |
| Afu8g01770 | putative GPI-anchored protein SwgA | 2.30 | 2.57 |
| Afu6g00510 | NADP-dependent alcohol dehydrogenase | 2.26 | 1.75 |
| Afu2g05140 | ankyrin repeat protein | 2.26 | 1.86 |
| Afu2g01140 | putative GPI anchored cell wall protein Dan4 | 2.25 | 1.83 |
| Afu4g13520 | oxidoreductase. short-chain dehydrogenase/reductase family | 2.15 | 2.21 |
| Afu4g11130 | conserved hypothetical protein | 2.16 | 2.43 |
| Afu1g03040 | conserved hypothetical protein | 2.16 | 1.73 |
| Afu5g01240 | putative general amidase | 2.15 | 1.89 |
| Afu3g10175 | hypothetical protein | 2.13 | 3.29 |
| Afu3g01120 | putative MFS multidrug transporter | 2.20 | 2.18 |
| Afu4g13070 | putative alpha/beta hydrolase | 2.10 | 1.62 |
| Afu3g07050 | putative WSC domain protein | 2.46 | 3.04 |
| Afu1g01900 | dienelactone hydrolase family protein | 1.98 | 1.89 |
| Afu6g03600 | putative integral membrane protein Pth11 | 2.16 | 2.61 |
| Afu6g08140 | cytochrome P450 monooxygenase | 1.96 | 2.33 |
| Afu4g09670 | conserved hypothetical protein | 1.95 | 2.40 |
| Afu5g00310 | putative flavin-containing monooxygenase | 1.94 | 1.91 |
| Afu4g13600 | putative C2H2 finger domain protein | 1.94 | 2.54 |
| Afu1g13240 | putative adenosine deaminase | 1.88 | 3.85 |
| Afu4g10170 | conserved hypothetical protein | 1.93 | 2.25 |
| Afu4g06170 | conserved hypothetical protein | 1.88 | 2.59 |
| Afu5g01620 | extracellular proline-rich protein | 1.87 | 1.57 |
| Afu2g17810 | putative RTA1 domain protein | 1.93 | 3.20 |
| Afu3g14130 | conserved hypothetical protein | 1.83 | 3.10 |
| Afu2g17460 | putative aldehyde dehydrogenase AldH12 | 1.84 | 1.82 |
| Afu4g08450 | conserved hypothetical protein | 1.84 | 2.41 |
| Afu1g13120 | conserved hypothetical protein | 1.84 | 3.89 |
| Afu6g13470 | conserved hypothetical protein | 1.81 | 1.65 |
| Afu3g05800 | serum paraoxonase/arylesterase family protein | 1.81 | 1.63 |
| Afu5g10290 | putative fructose-bisphosphate aldolase | 1.79 | 2.01 |
| Afu2g03270 | putative glycosyl hydrolase | 1.79 | 1.64 |
| Afu3g07560 | enoyl-CoA hydratase/isomerase family protein | 1.76 | 2.27 |
| Afu4g11150 | ADAM family of metalloprotease ADM-B | 1.76 | 1.75 |
| Afu4g00810 | hypothetical protein | 1.76 | 1.65 |
| Afu4g06980 | conserved hypothetical protein | 1.75 | 1.60 |
| Afu5g02000 | putative proline racemase | 1.75 | 2.50 |
| Afu8g07320 | putative endoglucanase | 1.75 | 2.89 |
| Afu3g01800 | GPI anchored dioxygenase | 1.75 | 2.10 |
| Afu2g15100 | conserved hypothetical protein | 1.74 | 2.07 |
| Afu4g11140 | putative DNA polymerase iota | 1.73 | 2.55 |
| Afu6g14060 | conserved hypothetical protein | 1.73 | 1.88 |
| Afu7g06640 | hypothetical protein | 1.73 | 1.70 |
| Afu5g01590 | putative geranylgeranyl pyrophosphate synthase | 1.72 | 1.74 |
| Afu6g10940 | conserved hypothetical protein | 1.72 | 2.55 |
| Afu8g07330 | putative isoflavone reductase family protein CipA | 1.71 | 3.74 |
| Afu4g01210 | hypothetical protein | 1.71 | 3.09 |
| Afu3g02360 | FAD dependent oxidoreductase superfamily | 1.70 | 1.52 |
| Afu2g00740 | conserved hypothetical protein | 1.66 | 1.61 |
| Afu7g06690 | putative flavin-containing monooxygenase | 1.64 | 1.58 |
| Afu4g01230 | putative GABA permease | 1.64 | 1.82 |
| Afu1g09300 | hypothetical protein | 1.63 | 2.46 |
| Afu7g02180 | UDP-N-acetylglucosamine pyrophosphorylase | 1.63 | 1.95 |
| Afu5g08250 | hypothetical protein | 1.63 | 2.21 |
| Afu6g02460 | putative glucosamine 6-phosphate acetyltransferase | 1.62 | 1.57 |
| Afu3g14165 | hypothetical protein | 1.61 | 2.21 |
| Afu2g14470 | putative oxidoreductase, FAD-binding | 1.60 | 1.70 |
| Afu2g00730 | FAD binding domain protein | 1.58 | 1.59 |
| Afu3g07570 | conserved hypothetical protein | 1.57 | 3.07 |
| Afu1g10420 | hypothetical protein | 1.56 | 1.63 |
| Afu1g02270 | putative ARS binding protein Abp2 | 1.55 | 1.71 |
| Afu5g08580 | alpha-1.6-mannosyltransferase subunit Och1 | 1.54 | 1.58 |
| Afu1g11560 | putative 4-hydroxyphenylpyruvate dioxygenase | 1.54 | 2.26 |
| Afu3g14150 | F-box domain protein | 1.54 | 2.52 |
| Afu5g09930 | conserved hypothetical protein | 1.53 | 1.58 |
| Afu8g00490 | putative PKS-like enzyme | 1.51 | 1.91 |
| Afu7g04980 | 3-ketoacyl-acyl carrier protein reductase | 1.51 | 1.83 |
| Afu3g09620 | putative RNA binding protein | 1.51 | 1.57 |
| Afu5g12220 | conserved hypothetical protein | 1.51 | 1.86 |
| Afu4g10200 | putative transcription factor RfeF | 1.50 | 1.93 |
| Afu6g02300 | putative serine/threonine protein kinase Kcc4 | 1.50 | 1.53 |
|  |  |  |  |
|  | **Wild type-specific FDGs at both time points** | **log_2_FC** | |
| **Identifier** | **Designation** | **1 h vs 0 h** | **3 h vs 0 h** |
| Afu2g08340 | conserved hypothetical protein | -3.55 | -3.77 |
| Afu1g10380 | nonribosomal peptide synthase Pes1 | -3.33 | -4.04 |
| Afu5g06660 | UPF0187 domain membrane protein | -3.19 | -1.58 |
| Afu4g08710 | putative short chain dehydrogenase | -3.14 | -2.45 |
| Afu3g13160 | conserved hypothetical protein | -3.02 | -2.89 |
| Afu3g09690 | putative extracellular thaumatin domain protein | -2.78 | -1.72 |
| Afu2g16340 | hypothetical protein | -2.73 | -1.74 |
| Afu8g01710 | putative antigenic thaumatin domain protein | -2.56 | -2.71 |
| Afu6g06530 | hypothetical protein | -2.44 | -1.77 |
| Afu6g10660 | putative ATP citrate lyase subunit Acl | -2.43 | -2.41 |
| Afu6g10650 | putative ATP citrate lyase, subunit 1 | -2.44 | -2.36 |
| Afu6g08240 | putative HEAT repeat protein DRIM | -2.43 | -1.54 |
| Afu7g04930 | alkaline serine protease PR1/allergen F18-like | -2.33 | -1.81 |
| Afu4g09590 | conserved hypothetical protein | -2.24 | -2.70 |
| Afu2g07740 | conserved hypothetical protein | -2.21 | -2.14 |
| Afu4g07030 | conserved hypothetical protein | -2.13 | -1.83 |
| Afu6g00620 | GPI anchored hypothetical protein | -2.13 | -2.78 |
| Afu5g07810 | DUF914 domain membrane protein | -2.08 | -1.55 |
| Afu1g10390 | putative ABC multidrug transporter | -2.97 | -3.11 |
| Afu4g14230 | putative MFS transporter | -2.05 | -1.89 |
| Afu2g03830 | allergen Asp F4 | -1.99 | -1.80 |
| Afu6g12360 | class I alpha-mannosidase 1A | -1.95 | -1.97 |
| Afu6g06535 | conserved hypothetical protein | -1.94 | -1.69 |
| Afu1g14800 | conserved hypothetical protein | -1.83 | -2.84 |
| Afu2g02440 | conserved hypothetical protein | -1.88 | -1.85 |
| Afu5g14320 | conserved hypothetical protein | -1.91 | -1.86 |
| Afu1g15830 | conserved hypothetical protein | -1.77 | -2.20 |
| Afu2g00660 | TcsB | -1.75 | -2.11 |
| Afu6g11090 | conserved hypothetical protein | -1.74 | -1.87 |
| Afu8g00780 | conserved hypothetical protein | -1.71 | -2.04 |

**Additional Table 3:** List of wild type-specific up- or down-regulated genes showing a similar differential regulation at both time points.

**Additional Table 4: Oligonucleotides used in this study**

| **qPCR** |  |  |
| --- | --- | --- |
| tubA qpcr-FOR | ACGGCGGAAACACGGAAAAC | Afu1g10910 |
| tubA qpcr-REV | CGAGATCGACCAGAACGGCA | Afu1g10910 |
| stl1 qpcr-FOR | ACAACGGTCTGCTACACTGG | Afu8g05710 |
| stl1 qpcr-REV | GCCCTCGAAACAGACAAGGA | Afu8g05710 |
| adh1 qpcr-FOR | ATGGTGGGGATGAGAAGCGG | Afu7g01010 |
| adh1 qpcr-REV | ACATACTCGGTCGCCTGCTG | Afu7g01010 |
| ags3 qpcr-FOR | TCAGTGTCTGGGGAGTGGGA | Afu1g15440 |
| ags3 qpcr-REV | GAGGCGGCGTGGTTATGTTG | Afu1g15440 |
| GF6P qpcr-FOR | TCATGGAACACCCTCTCGCC | Afu6g06340 |
| GF6P qpcr-REV | AACCGGAACCCCTTGCTCTC | Afu6g06340 |
| aglcnase qpcr-FOR | GCGGCCAATAGCACCTTCAC | Afu1g03352 |
| aglcnase qpcr-REV | AGAGGTCCGGGGTGTCTTCT | Afu1g03352 |
|  |  |  |
| **Amplification of flanking regions** |  |  |
| Adh1-up-FOR | TCCCAGAGCGGCTCCGCA | upstream region |
| Adh1-up-REV | ATGCCGGCCTGAGTGGCCGGTTGTTATCGGTGGGGCGAA | upstream region |
| Adh1-do-FOR | ATGCCGGCCATCTAGGCCATATCCTATCTTCGGTTGCTA | downstream region |
| Adh1-do-REV | GAGATTGCCTGAAGAGGTAAT | downstream region |
| GlcA-up-FOR | TCATAGATTGTTCTACTTACT | upstream region |
| GlcA-up-REV | AGGCCTGAGTGGCCTGTGCCGATTGCAGTGGGTTG | upstream region |
| GlcA-do-FOR | AGGCCATCTAGGCCCGACATCTCTTCCTATATATA | downstream region |
| GlcA-do-REV | CAACTATGATAGTCTATCATC | downstream region |
| Gtb3-up-FOR | GAATGGCTGCCACGCTGTT | upstream region |
| Gtb3-up-REV | GATGGTATGATTAGAGACTA | upstream region |
| Gtb3-do-FOR | ATCTATCTATCCTGCCCTAC | downstream region |
| Gtb3-do-REV | CTTTTACGAGGCGCACGAC | downstream region |
|  |  |  |
| **Amplification of the hygromycin cassette** |  |  |
| HygB-Kassette-FOR | CTCGGCCTAGATGGCCAT |  |
| HygB-Kassette-REV | TACCGGCCACTCAGGCCTA |  |
|  |  |  |
| **Detection of the targeted genes** |  |  |
| Adh1-FOR | ATGACTAAGTTCGACATCCCCT |  |
| Adh1-REV | TCATTCGGGCATCTCAAGGA |  |
| GlcA-FOR | ATGCGAAACCCTTTCGATCTGA |  |
| GlcA-REV | TTACTGTTGGTCCTCGGACT |  |
| Gtb3-intern-FOR | GTCATCACCCTCCACCATG |  |
| Gtb3-REV | AGTGTGCAAATTCAGGATTTC |  |

**Additional Table 4:** Oligonucleotides used in this study.
